# Accounting for uncertainty in computational models of ventricular tachycardia improves ablation guidance

**DOI:** 10.1038/s44161-026-00856-w

**Published:** 2026-08-14

**Authors:** Abdul Mateen Qadri, Ursula Rohrer, Fernando O. Campos, Iulia Nazarov, Ali-Razak Rashid, Pranav Bhagirath, C. Aldo Rinaldi, Ronak Rajani, Luca Azzolin, Aurel Neic, Gernot Plank, John Whitaker, Martin J. Bishop

**Affiliations:** 1 Research Department of Digital Twins in Healthcare, School of Biomedical Engineering and Imaging Sciences, King’s College London, London, UK; 2 Department of Cardiology, Guys’ and St. Thomas’ NHS Foundation Trust, London, UK; 3 Department of Cardiology, Amsterdam University Medical Center, Amsterdam, The Netherlands; 4 NumeriCor, Graz, Austria; 5 Division of Biophysics, Medical University of Graz, Graz, Austria; 6 BioTechMed-Graz, Graz, Austria

**Keywords:** Interventional cardiology, Biomedical engineering

## Abstract

Personalized ‘digital twin’ technology has the potential to revolutionize target guidance for catheter ablation therapy of ventricular tachycardia (VT); however, concerns regarding the practical implementation of these computationally intensive approaches, along with the robustness of simulation predictions given uncertainty in the interpretation and analysis of clinical imaging data used to reconstruct image-based models, are hindering clinical translation. Here we present a new technical framework that provides near-real-time guidance on VT ablation targets, inherently incorporating uncertainty in reconstructed scar anatomy. We demonstrate close agreement between simulated ECG ‘fingerprint’ signatures of VT circuits and clinical recordings in ischemic ablation patients, highlighting the need to account for variations in reconstructed scar anatomy to identify the best-matching simulated circuit. Finally, we introduce a robust method for integrating anatomical information across all viable simulated circuits from different model ‘instances’ into a simulated ablation target heatmap for practical clinical guidance.

## Abstract (web summary)

Qadri et al. develop a computational framework for near-real-time ventricular tachycardia ablation guidance that incorporates uncertainty in fibrotic scar reconstruction and simulates faithful ECG signatures of tachycardia circuits.

## Main

Catheter ablation is the treatment of choice for drug refractory ventricular tachycardia (VT) and results in the most durable arrhythmia suppression^1^; however, comprehensively identifying the arrthymogenic substrate remains challenging, often leading to prolonged procedures, increased complication risk and suboptimal success rates^2–4^.

Personalized in silico models derived from clinical imaging and electrophysiological data can be used to perform virtual VT induction protocols, visualize induced re-entrant circuits and predict relevant ablation targets^5–9^. These approaches offer pre-procedural insight, potentially guiding chamber access and reducing the need for VT induction during the procedure. By identifying target sites in advance, they may shorten mapping time, improving the safety profile of often lengthy, high-risk procedures^2^. Of note, models can reveal multiple potential circuits, which may enable more comprehensive targeting and potentially better outcomes, similar to image-guided strategies^10,11^. Previous studies have shown encouraging qualitative agreement with mapped VT circuits^8,9^ and clinical ablation targets^6^. More recently, simulation-identified regions have been shown to exhibit distinct electrogram characteristics, supporting their role in guiding substrate-based ablation strategies^7^.

Myocardial fibrosis is the key anatomical substrate for arrhythmia in structural heart disease, with scar architecture critically shaping re-entrant VT dynamics^12^. Scars can be assessed via pixel signal intensity analysis of late gadolinium-enhanced (LGE) cardiac magnetic resonance (CMR) or, in the case of chronic infarction, identification of abnormally thinned left ventricular (LV) wall from contrast-enhanced cardiac computed tomography (CCT)^8,9,13^. Identifying conduction channels within scar from detailed analysis of LGE CMR^10,11,14^ or CCT^13^ has shown promise for image-guided ablation strategies; however, applying fixed thresholds to scar delineation, whether based on intensity or wall thickness, fails to account for patient-specific variability in imaging, contrast uptake and segmentation approaches^15,16^, as well as population and disease-specific LV wall thicknesses (LVWTs)^17^. This introduces important uncertainty in the processing, analysis and interpretation of clinical imaging data used to define scars in patients with structural heart disease. The resulting variability in reconstructed three-dimensional (3D) scar anatomy has been shown to affect both VT inducibility and circuit dynamics^18–20^ in personalized models. Consequently, reliance on a single-patient model, with potentially inaccurate scar representation, poses a major challenge for using in silico approaches to guide ablation.

A major limitation of existing in silico approaches for VT ablation guidance is their high computational cost due to reliance on reaction-diffusion models. Execution of the virtual induction protocols required to identify VT circuits and ablation targets in each patient model can take up to 24–48 h on external high-performance computing facilities (HPC)^21^. This restricts practical clinical integration and prevents systematic exploration of uncertainty, which would require multiple model runs. To address this, we developed the Virtual Induction and Treatment of Arrhythmias (VITA), a near-real-time method that comprehensively identifies potential VT circuits within a personalized model in ~20 min on a standard desktop^19,21,22^. VITA reproduces anatomically consistent VT circuits compared to conventional reaction-diffusion approaches, but with orders of magnitude faster computation. Crucially, this efficiency enables incorporation of uncertainty in personalized models. While VITA has successfully shown utility in stratifying arrhythmic risk in different clinical situations^19,22^, the VT circuits themselves predicted by VITA have not yet been comprehensively validated against clinical data.

The 12-lead ECG is the primary noninvasive tool for assessing arrhythmias; however, no previous simulation study has directly compared simulated ECGs from model-predicted VT circuits with the clinical 12-lead VT recordings. This represents a critical step for validating in silico predications and building clinical confidence to support translation of these promising technologies into practice.

The goal of this study is to comprehensively evaluate VITA-predicted VT circuits from CT-derived personalized models by directly comparing simulated 12-lead ECG signatures with clinical VT recordings in patients with ischemic cardiomyopathy (ICM) undergoing ablation. A central feature is the incorporation of uncertainty in clinical imaging data used to define scar. By sweeping across a range of CT-derived LVWT thresholds, multiple model ‘instances’ with varying scar architecture are generated, enabling systematic comparison with clinical arrhythmias to identify the model that best reflects the observed VT. Building on this, we introduce a new ablation target guidance heat map (Simulated Ablation Target HEat (SATHe) Map), which integrates all simulation outputs into a clinically actionable tool. Across the cohort, simulated targets showed strong agreement with mapped VT circuits and surrogate markers, highlighting the potential of this framework to enhance ablation planning and execution.

## Results

**Fig. 1:**
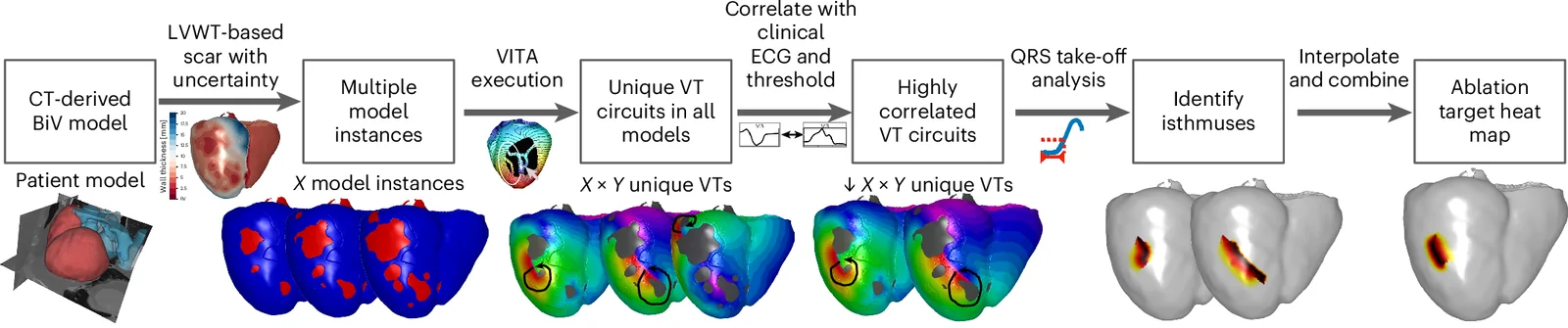
Pipeline showing generation of the ablation target heat map. BiV models are created from segmented CCT images. Different thresholds of LVWT are used to define chronic scars to create multiple model ‘instances’ for each patient. Application of our rapid re-entrant circuit path-finding approach (VITA) uncovers multiple uVT circuits in each model instance, with simulation of corresponding ECG signatures. Correlation of simulated ECGs with the clinical target ECG VT template identifies highly correlated VT circuits. Corresponding isthmuses of these highly correlated circuits are spatially combined to generate the SATHe Map, intrinsically incorporating uncertainty in scar definition from clinical images.

**Extended Data Fig. 1:**
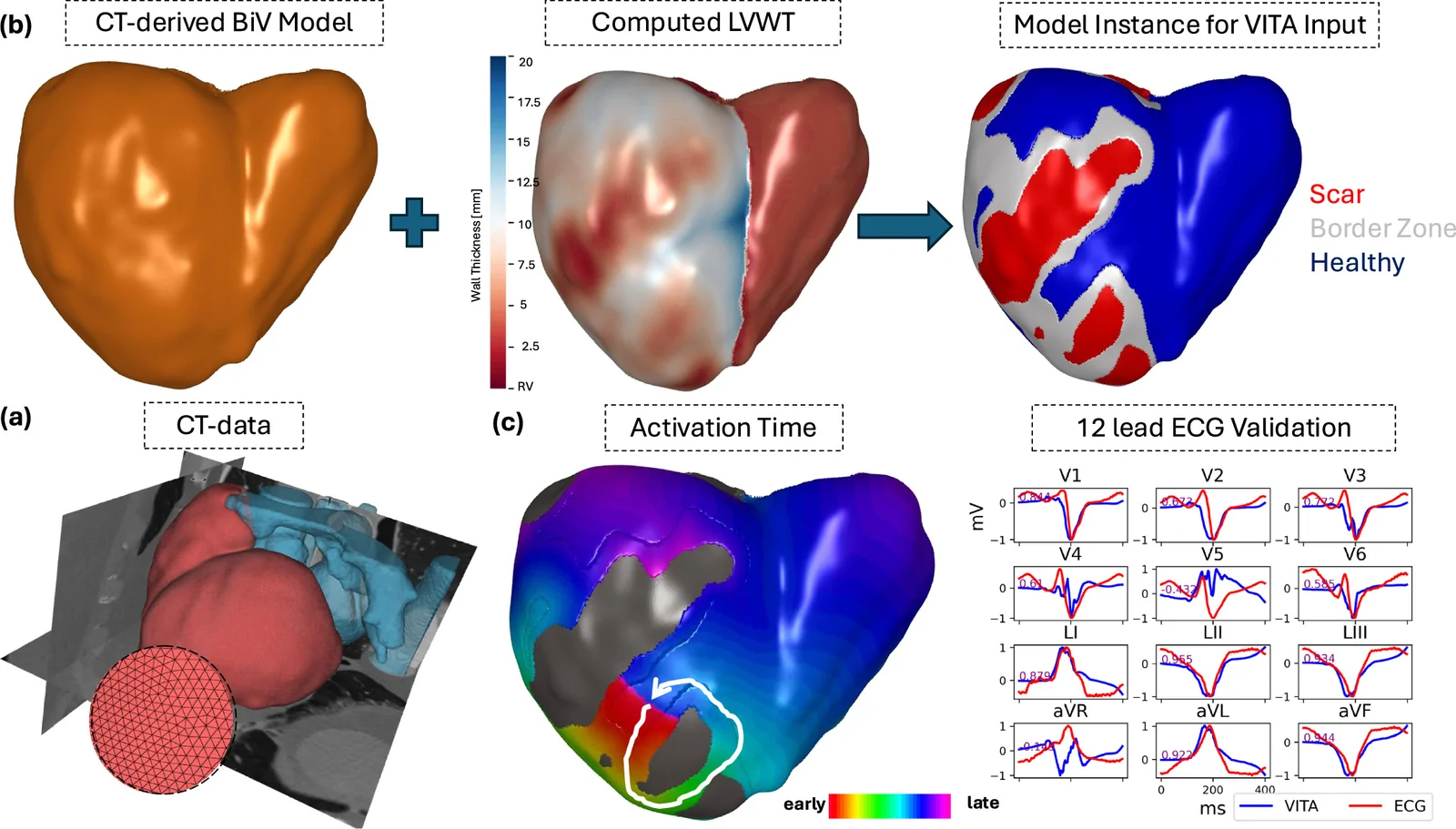
Computational workflow. Computational model generation and identification of relevant VT circuits. (**a**) Illustration of tetrahedral finite element mesh of BiV geometry. (**b**) Using LVWT for substrate characterization and generating model instances. (**c**) Identification of relevant VT circuit by correlating with clinically induced VT.

Figure 1 summarizes the workflow of this study. In brief, CT-based LVWT is used to create multiple model ‘instances’ of different reconstructions of scar substrate, conduct rapid simulations to identify viable VT circuits, and correlate their ECG signatures with a clinical target template. The simulated ablation target heatmap is then constructed by combining key features from circuits that most closely match the target clinical VT. The model generation process is summarized in Extended Data Fig. 1.

### Implication of uncertainty in scar threshold and VT inducibility

**Fig. 2:**
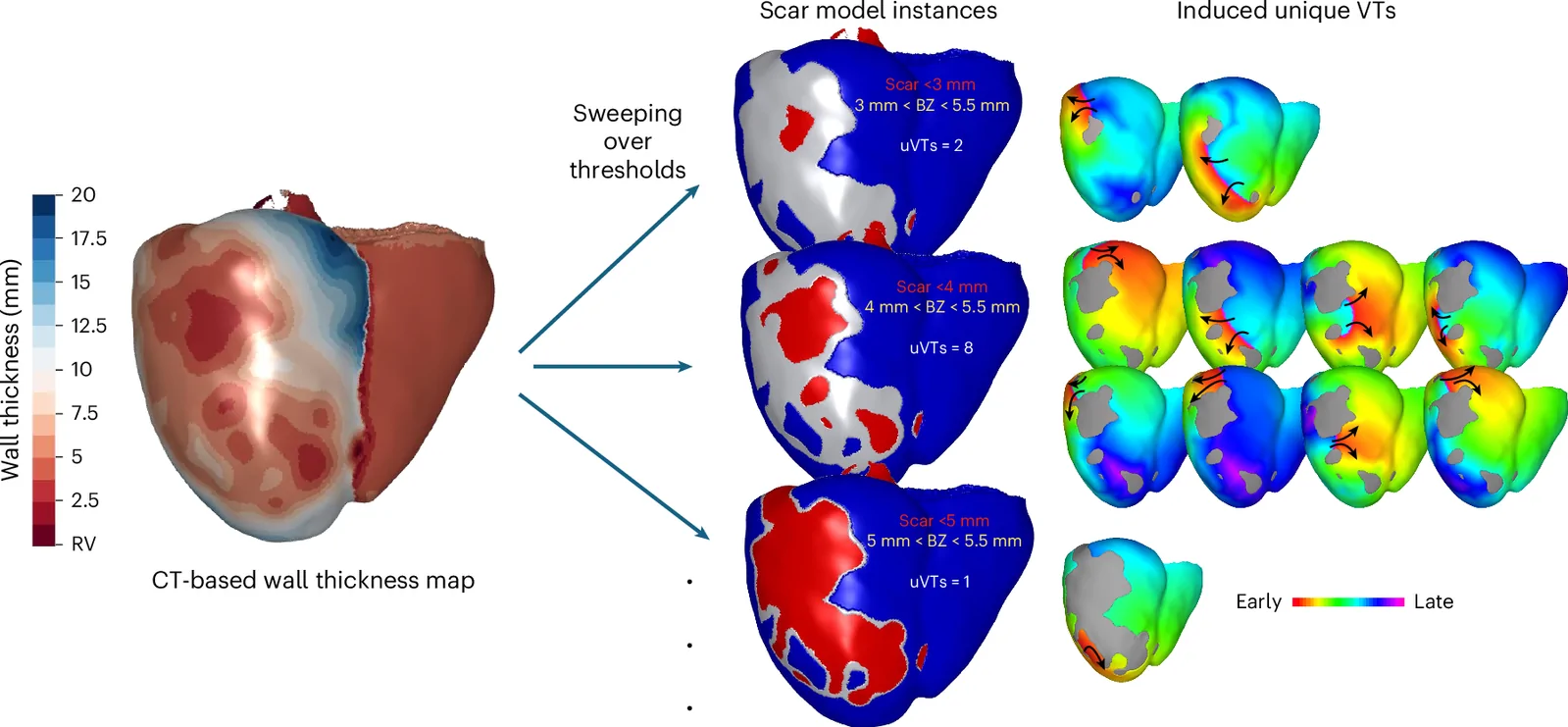
Example of scar thresholding in a representative patient. LVWT map derived from CT-based model (left). Three example model instances formed by selecting scar thresholds at LVWT < 3 mm, <4 mm and <5 mm producing markedly different scar morphologies (center). uVT circuits uncovered in each model instance (scar shown in gray) demonstrating the substantial variability in arrhythmogenic substrate between model instances (right).

To assess the impact of uncertainty in scar characterization derived from CT imaging data, we first generated multiple model instances for each patient by applying different LVWT thresholds to define dense scar and border zone (BZ) tissue (Methods, ‘Computational model generation’ section). Figure 2 illustrates this process for a representative patient. The panel on the left shows the continuous LVWT map derived from CT, with thinner regions corresponding to areas of chronic myocardial scar. In the center, three example model instances are shown, reconstructed using thresholds of <3 mm, <4 mm and <5 mm to delineate scar. As the threshold was varied, the resulting scar morphology changed substantially, leading to clear visual differences in the arrhythmogenic substrate available to sustain re-entry.

For this particular patient, the <3 mm threshold produced a limited scar region that supported only two unique VT (uVT) circuits, shown in the right panel. At <4 mm, the scar expanded, uncovering a more complex architecture with multiple isthmuses capable of sustaining re-entry, resulting in eight uVTs. By contrast, further increasing the threshold to <5 mm caused coalescence of scar regions and loss of critical channels, reducing inducibility to a single VT. This non-monotonic relationship between LVWT threshold and the number of inducible VTs highlights how small changes in scar definition can fundamentally alter the electrophysiological behavior of the model. This also underscores the necessity of incorporating uncertainty analysis into personalized predictions, as reliance on a single threshold risks overlooking clinically relevant circuits.

**Fig. 3:**
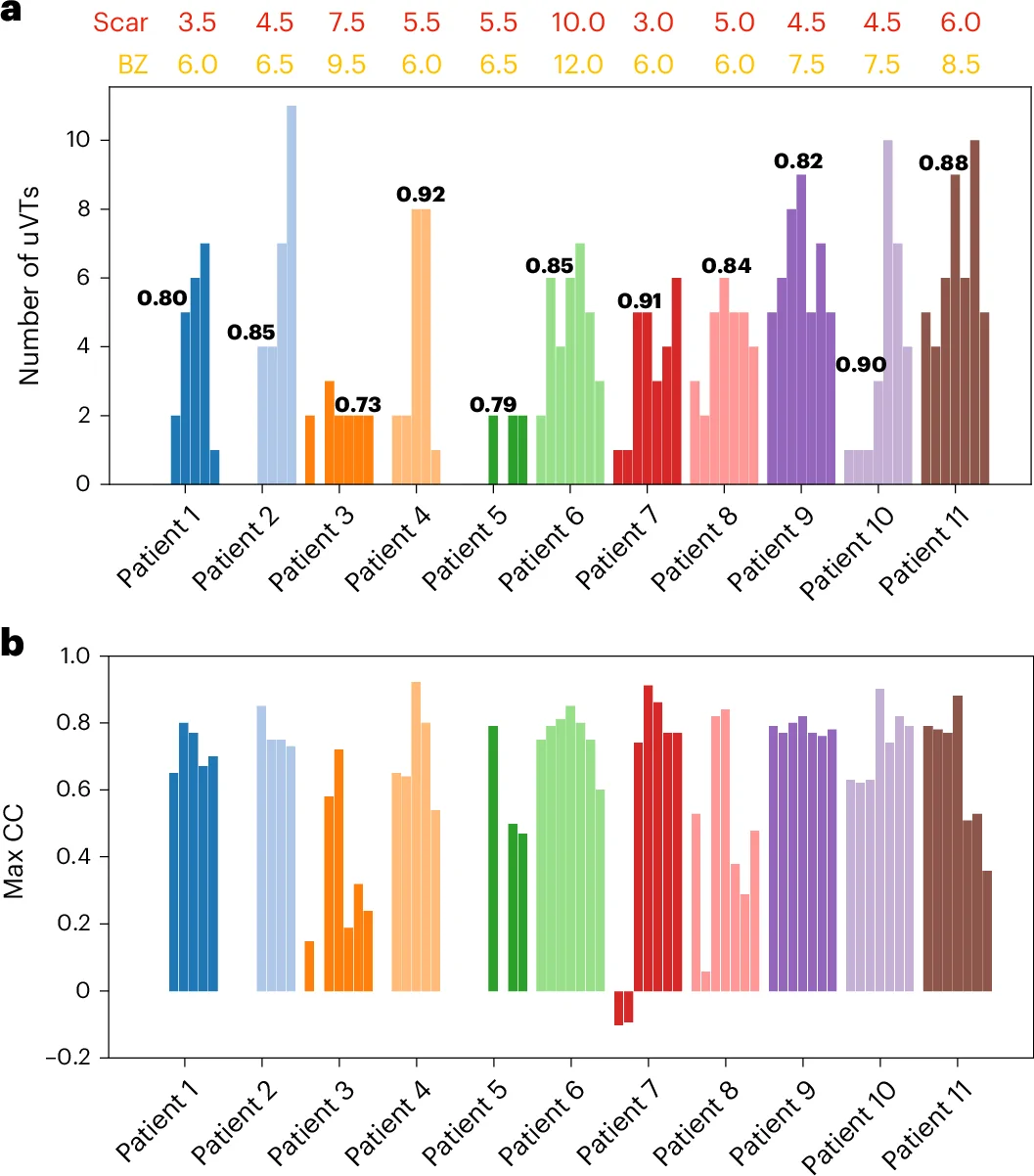
Distribution of induced uVTs and the maximum correlation with the clinical VT across model instances for all patients. **a**, Number of uVTs detected across scar-BZ model instances for 11 patients. Bars correspond to scar thresholds in 0.5-mm increments around the threshold yielding the peak CC. Three increments of scar threshold above the model instance yielding the peak CC and three below the peak are displayed. The central bar representing the model instance with the peak CC showing the value of the CC matching the clinical VT above it. The corresponding specific scar-BZ threshold levels for this model instance are shown in rows at the top (red, scar; yellow, BZ). Note: some model instances yielded zero uVTs, specifically: 2, 3, 1, 2 and 4 for patients 1–5, respectively. Patients 6–11 had no zero uVTs for any model instance shown. **b**, Similar plot to **a**, showing instead the explicit max CC values obtained within each model instance with the clinical VT target. Source data

To further examine the relationship between scar definition and VT inducibility across the entire patient cohort, we plotted the number of uVTs identified across scar-BZ model instances in all 11 patients with ICM (Fig. 3a). In later analysis, we further quantified agreement between simulated and clinical VTs by calculating correlation coefficients (CCs) for ECGs from all simulated circuits across all model instances against the presenting clinical VT ECG trace (Methods, ‘Comparison of simulated and clinical ECGs’ section). For each patient, three scar thresholds below and three above the threshold corresponding to the model instance producing the VT with the peak CC matching the clinical VT are displayed in 0.5-mm increments, allowing local sensitivity to substrate definition to be visualized. Across all scar thresholds, patients exhibited a median of 26 uVTs (range 6–45), corresponding to a median of 3 uVTs per patient per scar threshold (range 0–11) model instance.

Several important observations emerge. First, different scar/BZ thresholds uncovered different uVT distributions in different patients. Second, the scar instance yielding the highest CC did not consistently correspond to the highest number of uVTs. This was the case for patients 1–3, 6, 7, 10 and 11 highlighting that the best ECG match was not necessarily obtained at the most arrhythmogenic substrate configuration. Third, relatively small changes in scar threshold used to create model instances could lead to dramatic differences in the number of detected uVTs in that model instance (as shown in Fig. 2). For example, in patient 1 the number of uVTs decreased sharply from seven at SCAR 4.5 mm to a single VT at 5.0 mm.

Notably, in some cases, only a very narrow range of scar thresholds permitted detection of any VTs at all, with, in some cases, no VTs being inducible either side of the peak CC scar threshold. For patient 2, at the peak CC scar threshold (4.5 mm), lowering the threshold to 4.0 mm resulted in no inducible circuits. These findings underscore the high sensitivity of VT inducibility to subtle changes in scar reconstruction.

Finally, across patients there was no monotonic relationship between increasing scar threshold and the number of inducible VTs. Instead, the number of uVTs fluctuated in a nonlinear manner, reflecting the complex interplay between scar extent, channel formation and the preservation or collapse of re-entrant isthmuses (highlighted in Fig. 2). This behavior reinforces the importance of performing uncertainty analysis across a range of scar thresholds, as reliance on a single definition risks either overestimating or underestimating arrhythmogenic potential.

### ECG correlation analysis of simulated VT circuits against clinical ECGs

Not only did the number of induced uVTs vary substantially across different scar threshold model instances (shown in Fig. 3a), but so did the maximum CC of the resulting ECGs, shown in Fig. 3b. Similar to uVTs, maximum CC values showed no consistent trend with scar thresholds. Notable variation in maximum CC was often seen upon slight changes (just ±0.5 mm) in scar threshold between adjacent model instances (for example, patient 8).

Across the cohort, all patients demonstrated at least one simulated VT with a strong match to the clinical VT (maximum CC range 0.75–0.92, median 0.85). The highest correlation was observed in patient 4 (max CC = 0.92 at thresholds of SCAR 5.5 mm, BZ 6.0 mm), followed by patient 7 (0.91 at SCAR 3.0 mm, BZ 6.0 mm) and patient 10 (0.90 at SCAR 4.5 mm, BZ 7.5 mm). Patient 3 showed the lowest match (0.75 at SCAR 7.5 mm, BZ 9.5 mm), noted to be at relatively high thresholds to delineate scar from LVWT.

**Fig. 4:**
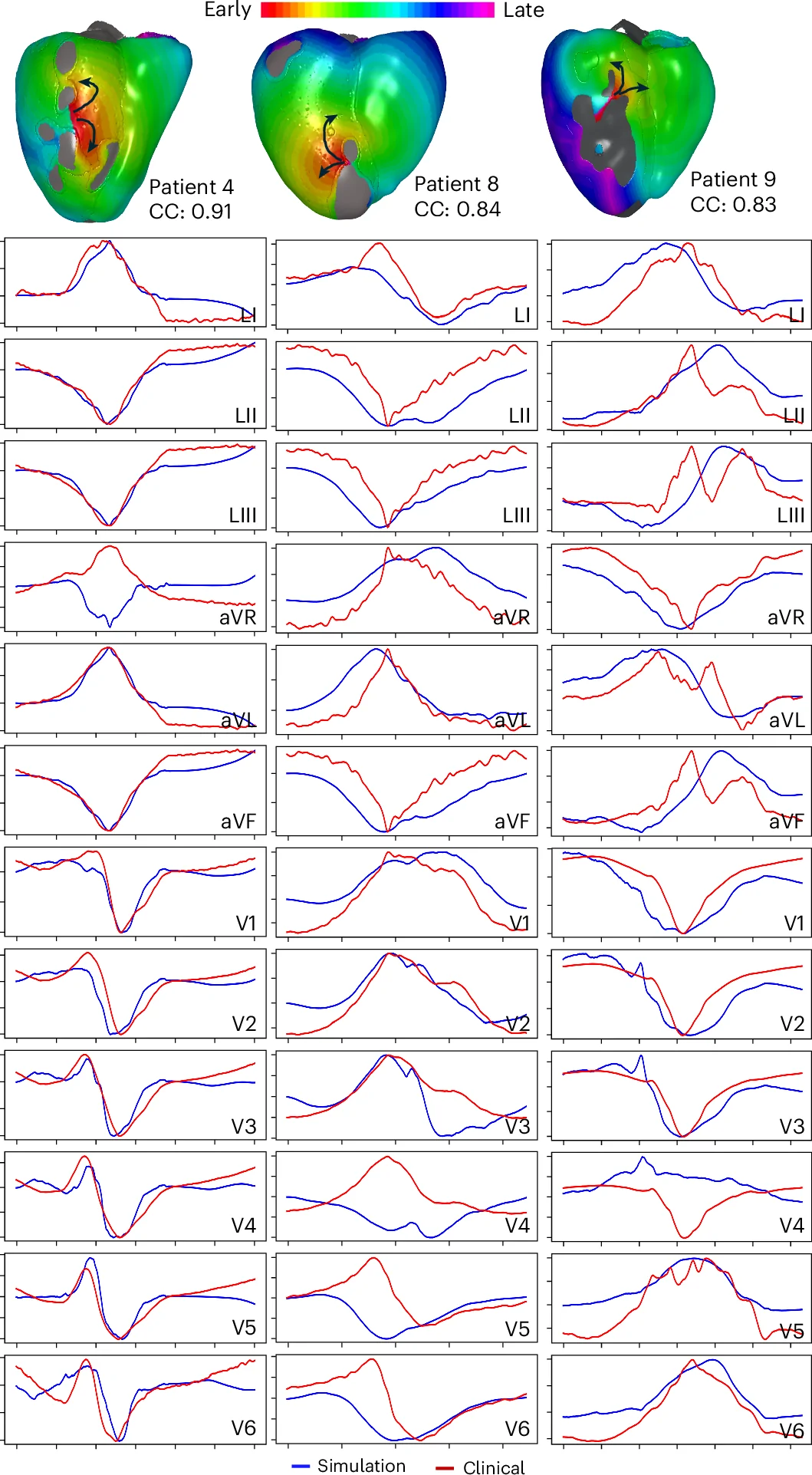
Simulated activation maps and ECG comparisons for three patients at maximum CC value. Simulated VT circuits and corresponding activation maps are shown alongside overlays of simulated (blue) and clinical (red) ECGs. Mean CC values reported correspond to averages over the top ten traces (as described in Methods, ‘Quantitative ECG comparison’ section).

Figure 4 presents simulated local activation time (LAT) maps and corresponding ECGs for three representative patients, each showing the simulated uVT that most closely matched the target clinical VT (the case with the maximum CC value). For each patient, the model structure with scar distribution and simulated circuit with re-entrant pathway is shown alongside the simulated 12-lead ECG overlaid with the clinical VT trace obtained during invasive electrophysiological testing.

Patient 4 showed good agreement across all 12 leads, with an overall CC of 0.92. Patient 8 showed good agreement in leads V1–V3, with reduced match in V4–V6 (mean CC of 0.84). Patient 9 achieved a mean CC of 0.83, displaying matching polarities across all leads (except in V4) with notching effects (not present in the simulations) slightly attenuating the mean CC value.

Overall, these examples highlight the ability of personalized simulations to not only reproduce surface ECG features of clinical VT with high fidelity, but also the importance of incorporating uncertainty in facilitating these close matches, supporting their potential utility in guiding targeted ablation strategies.

### Incorporating uncertainty in ablation target heat map guidance

**Fig. 5:**
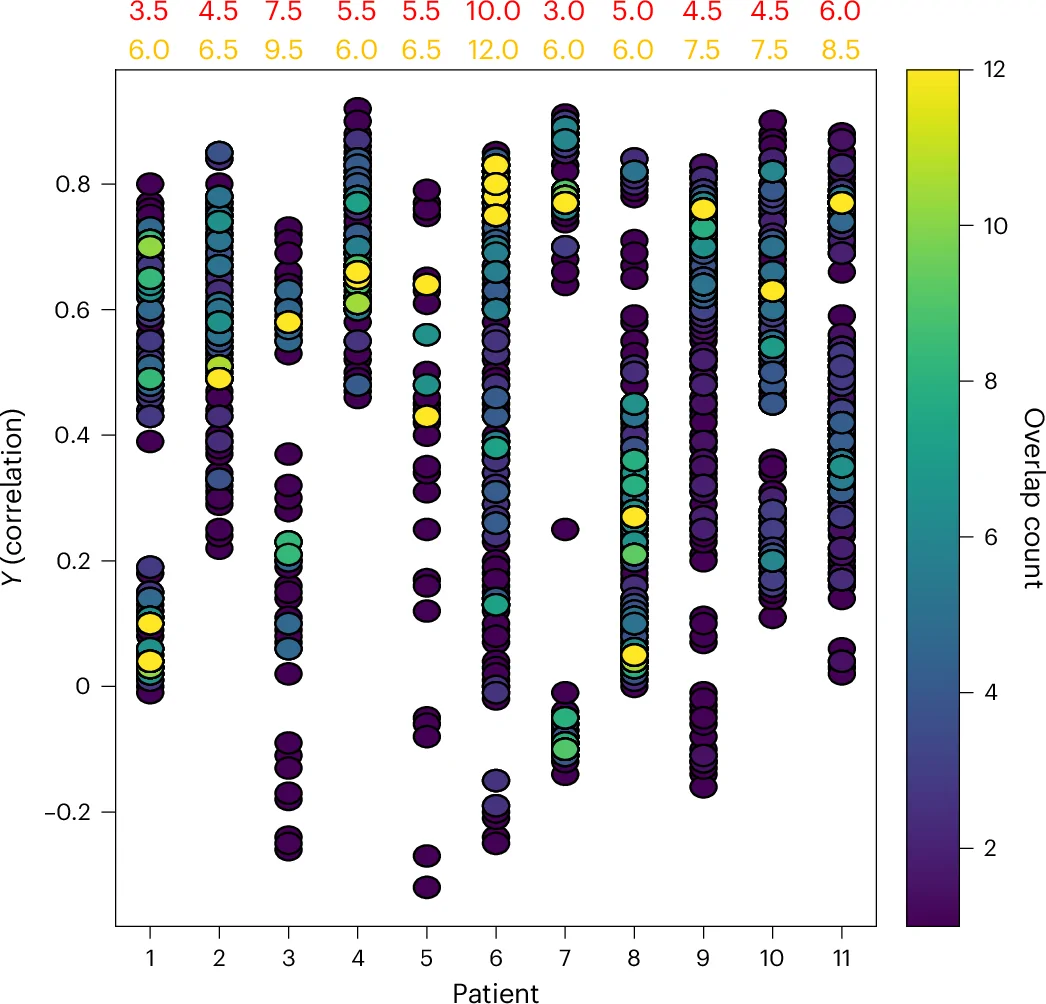
Scatter-plot of CCs for all simulated uVTs across all scar-BZ model instances in all patients. Each point denotes the CC for a uVT against the clinical ECG target. Maximum CCs across patients range from 0.75 to 0.91. As in Fig. 3, the scar-BZ threshold model instance yielding the max CC is annotated for each patient, and overlap counts (color bar) indicate the number of distinct uVTs sharing similar CCs. Source data

As shown in Fig. 3a, accounting for all model instances with variations in scar substrate definition uncovered multiple VTs in each patient. In addition to the most closely matching circuits (Fig. 4), many other uncovered VT circuits had relatively high CCs that closely matched the clinical VT, whereas others represented circuits which correlated less well. Figure 5 shows a scatter-plot of CCs for all uVTs against the clinical VT for that patient. Distinct clustering of CC values (shown by the color bar) was observed in some patients, reflecting groups of VTs with similar morphologies and thus correspondingly similar CCs. Of note, patients 6, 7 and 9–11 showed a particularly high density of circuits (yellow colors) with CC > 0.75, indicating multiple overlapping VTs capable of reproducing the clinical morphology with high fidelity. Multiple circuit configurations within the substrate may, thus, equally account for the clinical VT presentation, further underscoring the value of uncertainty analysis to robustly identify clinically relevant ablation targets.

### Comparison of SATHe Map predictions with clinical measurements

**Fig. 6:**
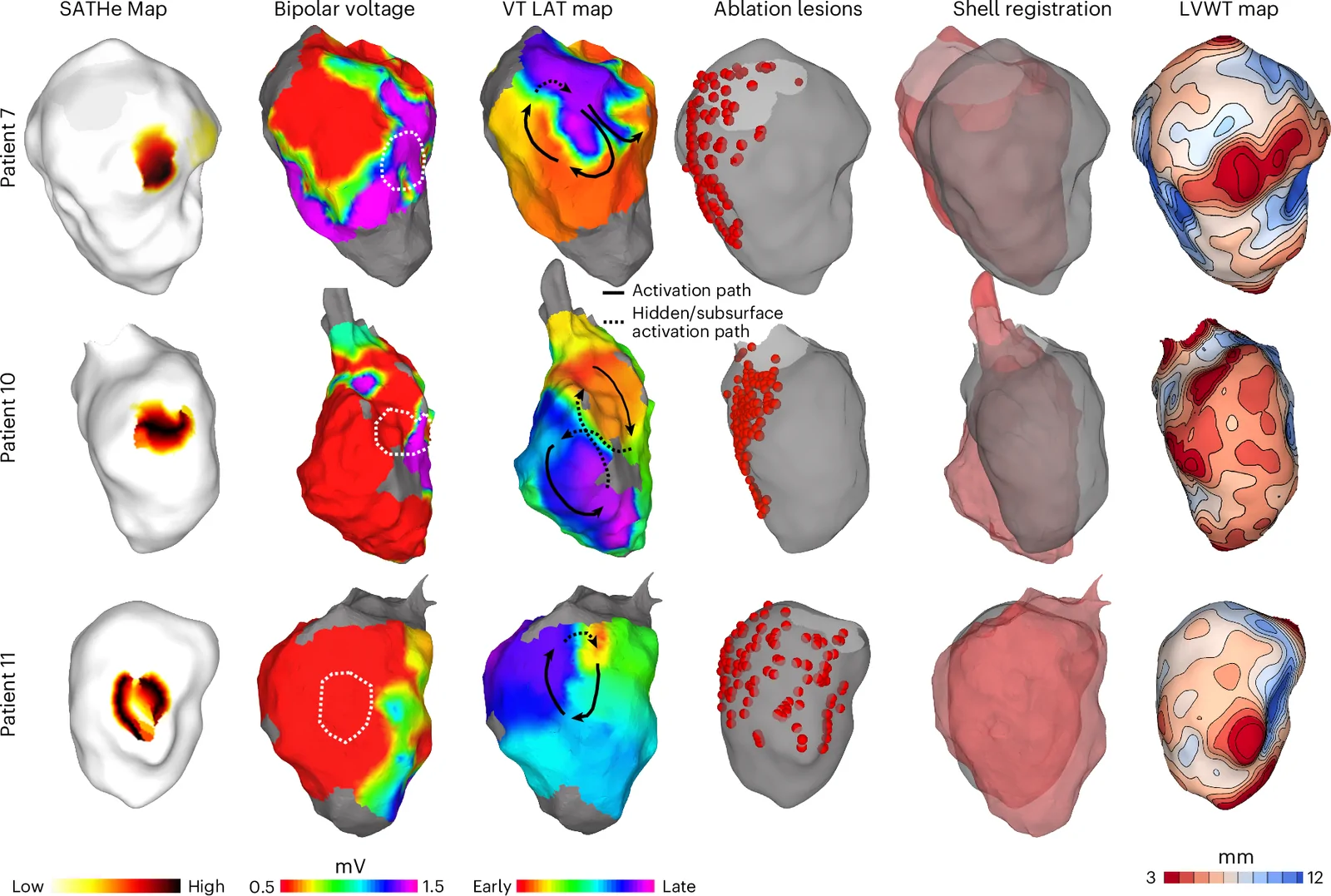
Comparison of SATHe Maps with bipolar voltage map, ablation lesions and LAT maps of VT circuits for three patients. Bipolar voltage and VT LAT maps are plotted on CARTO shell geometries following registration with computational model using ultrasound-derived anatomical fiducials (Methods, ‘Comparison with clinical data’ section), shown in the shell registration panel. LVWT maps (far right) are plotted on the LV endocardial model surface. White dashed highlighted areas in bipolar voltage maps show approximate locations of hotspot areas in SATHe Maps following registration between geometries. Limits on the bipolar voltage maps highlight dense scar in red (<0.5 mV) and healthy myocardium in purple (>1.5 mV). Black arrows in VT LAT maps show approximate locations of diastolic isthmuses delineating the activation pathway (solid line) and hidden/subsurface activation pathway (dashed line) characteristic of transitions between early-to-late activation in close spatial proximity.

As shown in Fig. 5, multiple uVT circuits, with slight morphological differences but similarly close matches to the clinical VT template, can be uncovered across model instances for each patient. Amalgamation of the anatomical information regarding these similar uVT circuits was used in the construction of the SATHe Maps (Methods, ‘Construction of simulation prediction heat map for ablation target guidance’ section). Figure 6 shows SATHe Maps for three patients where the gold-standard of LAT mapping of the VT circuit was performed. Here, the SATHe Map (left) is compared against bipolar voltage amplitude alongside the acquired VT LAT map (both plotted on the electro-anatomical mapping (EAM) shells following registration to the model) and ablation lesions (mapped onto the model surface), with the LVWT map also shown (far right). Note that in all cases, ablation lesions were delivered to disperse regions, using a largely substrate-based approach. Across the three patients, SATHe Maps highlight focused regions, which closely colocalize with the diastolic isthmus in the LAT map (highlighted by arrows), demarcating characteristic transitions between early-to-late activation (red to blue) in close spatial proximity (directly encompassed in patients 7 and 10, whereas on the periphery in patient 11). These regions are generally also located at the periphery of dense scar (identified by bipolar voltage). Specifically, patients 7 and 10 show hotspots directly encompassing regions of intermediate voltage amplitude (immediately proximal to a large area of low voltage), characteristic of VT exit sites, whereas patient 11 the hotspot is slightly to the left of such areas. SATHe Map hotspot regions also colocate with areas at the periphery of chronic wall-thinning (right) in all three patients.

**Fig. 7:**
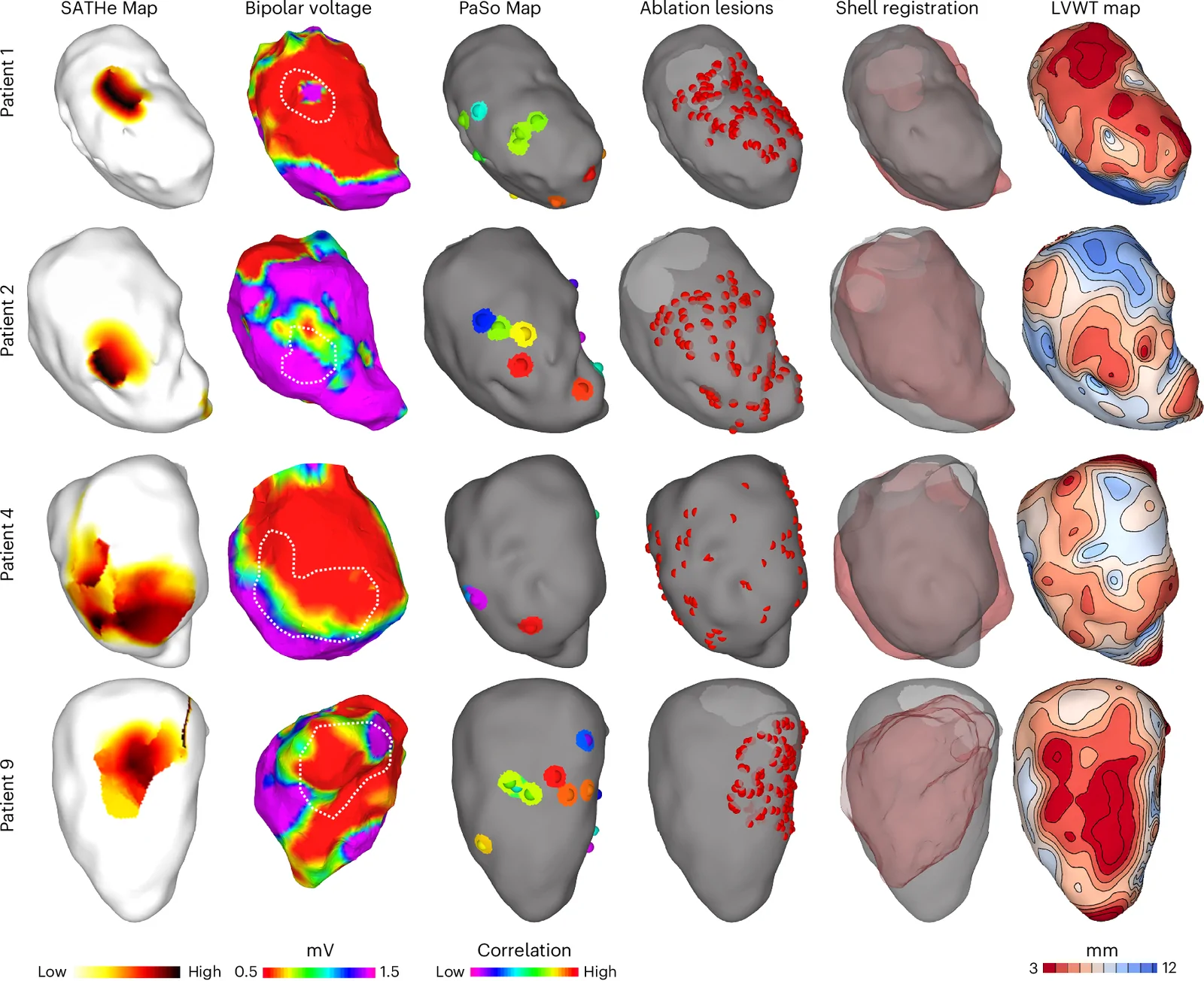
Comparison of SATHe Maps with bipolar voltage map, ablation lesions and PaSo Maps for four patients in whom VT mapping was not possible. Bipolar voltage, lesions, registration and LVWT maps are the same as in Fig. 6. PaSo points are plotted following mapping back on to the computational model with colors indicating the pace-map correlation matching the target clinical ECG.

For those patients in whom VT mapping was not possible, Fig. 7 shows a similar comparison with PaSo Maps acquired following pace mapping. As in Fig. 6, the predicted hotspot from the SATHe Map corresponds to areas at the periphery of dense scar. Patients 2, 4 and 9 show good correlation between the SATHe Map generated against highly correlated pace mapping points, along with overlap with the ablation lesions. In patient 1, the SATHe Map hotspot shows good overlap with ablation lesions.

**Fig. 8:**
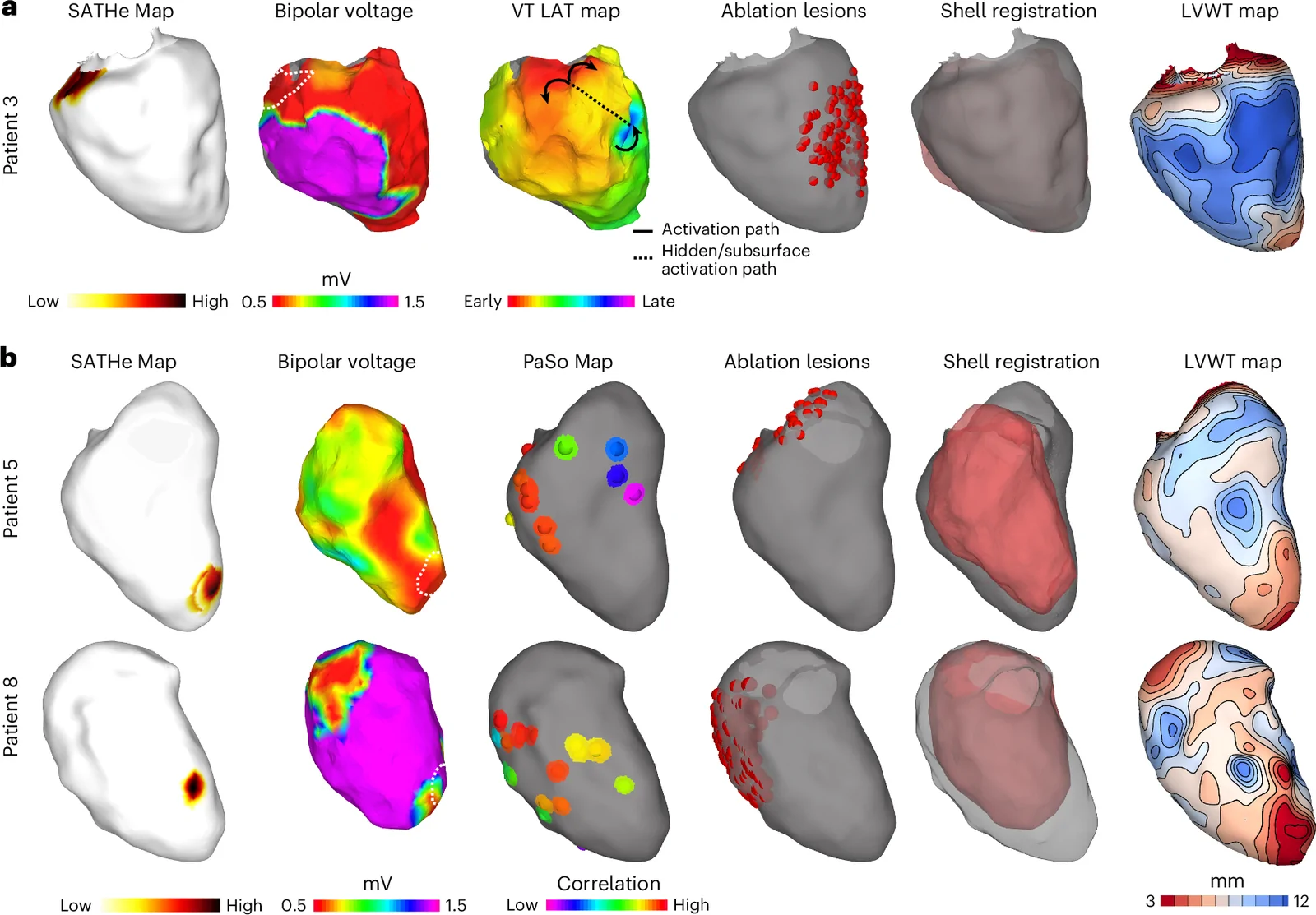
Comparison of SATHe Maps with clinical data in the three patients with relatively little pathologically thinned LV wall. **a**, Patient 3 comparison with mapped VT circuit, similar to Fig. 5. **b**, Patients 5 and 8 comparison with PaSo Maps, similar to Fig. 7.

In patients 3, 5 and 8 the comparison with clinical data of VT site-of-origin is less strong, shown in Fig. 8. Here, the SATHe map prediction for patient 3 is compared against an LAT VT circuit map, whereas in patients 5 and 8, VT mapping was not possible and the PaSo Map is used. Such comparisons help elucidate the mechanisms underlying these discrepancies, particularly in relation to the LVWT maps shown in Fig. 8 (right). In contrast to other patients (Figs. 6 and 7), the three particular patients in Fig. 8 show very little pathologically thinned LV wall, with almost no regions <4–5 mm except in the apical areas where physiological thinning may be expected. Specifically, LV wall-thinning <6 mm was present in <10% of their LV (mean 5.4%, range 3.5–7.4%). In contrast, all patients in whom the model provided guidance that matched closely with ablation targets (Figs. 5 and 6) consistently had >10% LV with wall-thinning <6 mm (mean 19.6%, range 11.7–30.2%). In patients 5 and 8, the SATHe Map hotspot is predicted close to the apex, whereas in patient 3 it is near the base, evidently found as channels open up in these areas as LVWT scar thresholds are changed. However, the simulated VT circuits uncovered in these regions showed relatively poor matches with the target clinical VT ECG, with Fig. 5 showing relatively few uVTs with high CCs. Note that patient 6 is not shown, as no ablation was performed.

#### Utility of model in predicting non-inducibility

Finally, the simulation pipeline was separately also applied to a subgroup of three patients (with non-ischemic cardiomyopathy; NICM) who were non-inducible for VT upon programmed electrical stimulation (Methods, ‘Clinical data’ section). As expected, the VITA algorithm failed to uncover any VT circuits in any of the generated model instances for these patients. Analysis of LVWT maps for these patients showed no areas of pathological thinning, in keeping with their lack of inducibility. All three patient models had <1% of their LV with LVWT < 6 mm.

## Discussion

We present a framework for integrating intrinsic uncertainty in CT-derived scar reconstruction into in silico models to predict critical re-entrant VT sites in patients with structural heart disease undergoing VT ablation. The demonstration of an efficient approach to incorporate uncertainty lays the foundation for wider application of uncertainty-aware model parameterization. In doing so, we have shown that small changes in CT-derived scar thresholds markedly alter the number and characteristics of predicted re-entrant circuits. We also directly compare simulated and clinical VT ECGs, showing that the closest matches are only uncovered when uncertainty is accounted for. By considering the range of predicted arrhythmias and their robustness to parameter changes, we present an approach to quantify prediction confidence to construct an ablation target heat map (SATHe Map) suitable for clinical workflow integration.

### Importance of considering uncertainty

Previous simulation studies have shown that image resolution used for model construction^18^, scar 3D interpolation methods^20^, along with pixel intensity thresholds for LGE scar segmentation^19^ directly affect scar mass and, critically, morphology, particularly the presence of conducting isthmuses that drive VT dynamics. Such changes in scar reconstruction can determine whether a model is inducible under identical protocols^18^, and influence both the number and dynamics of induced VTs^18–20^. This variability in simulated model predictions highlights the need for caution when relying on a single personalized model to guide ablation targets.

This study aimed to develop a framework to explicitly account for the inherent uncertainty in all clinical and imaging data. Here, we evaluated how varying CT-based scar thresholds affects model outputs, and demonstrated a computationally efficient framework for generating multiple instances to capture this uncertainty. By using each model instance within our VITA framework^21^, we rapidly assessed potential VT circuits in near-real time, not possible with conventional direct reaction-diffusion simulations, which can take 1–2 days per model on external HPC systems^21^.

We showed that each model instance can support multiple potential VT circuits (Figs. 3 and 5), with circuit counts peaking at LVWT thresholds slightly higher (~<5 mm) than the conventional <2–3 mm used to define ischemic scar^8,9,13^. By sweeping across thresholds and simulating ECGs for each circuit, allowed us to assess their relevance to the clinical VT. This uncertainty-driven approach enabled identification of the circuit with the highest correlation to the target ECG. The strong sensitivity of these correlations to small threshold changes (Fig. 3b), highlights the need to account for uncertainty in image-based scar reconstruction and cautions against a one-size-fits-all LVWT threshold. Without uncertainty analysis, the best-matching circuit may be missed, often occurring outside conventional LVWT thresholds used to define chronic ischemic scar^8,9,13^ (Figs. 2 and 5).

In several cases, varying the scar threshold by as little as 0.5 mm rendered VT non-inducible, underscoring the sensitivity of predictions to minor changes in substrate definition. This exposes a key limitation of single-threshold approaches, which may miss clinically relevant VT circuits if the chosen threshold is even slightly mis-specified. By systematically incorporating this variability, our framework provides a more robust characterization of the arrhythmogenic substrate.

Accounting for uncertainty also reflects the realities of clinical practice, where image and electrical measurements all introduce inherent variability. By evaluating a spectrum of substrate definitions reveals regions of consistent arrhythmogenic potential across multiple model instances, identifying more robust ablation targets. This comprehensive approach supports the translation of personalized models into clinically actionable, decision-support tools.

### Validation of VT circuit ECG signatures

Previous simulation studies have compared VT circuits with clinical recordings^6–9^, although largely through qualitative visual comparisons of simulated VT dynamics with clinical activation time maps of stable VT circuits^6,8,9^, which are difficult to obtain across full cohorts. Here, we instead quantify agreement by correlating simulated and recorded ECG signatures for the entire cohort, something not attempted in any other VT simulation study to date. Across all 11 patients with ICM with inducible VT, our approach achieved a mean CC of 0.845, with all patients >0.75, and three >0.9.

Given the inherent simplified nature of computational models, slightly lower CCs than clinical benchmarks (for example pace mapping where >0.95 is generally used) would be anticipated. Nevertheless, the consistently high correlations across our cohort, together with strong lead-specific visual agreement, demonstrates that CT-based scar models can predict clinical VT with high fidelity (Fig. 4). This is further supported by concordance between the heat maps with the ablation targets or mapped VT circuits (Figs. 6 and 7), providing further clinical confidence in the in silico predictions. While further model personalization (such as His Purkinje System modeling or conductivity tuning^23^) is possible, clinical translation requires balancing added model complexity against practicality, particularly if additional clinical data acquisition is required.

Our approach requires additional torso imaging and localization of ECG electrodes to accurately recover extracellular potentials. Such imaging is increasingly part of standard pre-ablation workflows in many centers. Prospectively, electrode positions could be captured using 3D camera systems already employed in platforms such as Corify (Corify Care) or VIVO (Catheter Precision).

### Comparison with clinical VT EAM data

By transparently comparing model predictions with clinical mapping data across the cohort, we show the robustness of this approach for guiding VT ablation in diverse pathologies. Where VT circuits were directly mapped, SATHe Map hotspots closely matched the identified isthmus. In most other cases, strong agreement was observed with surrogate markers of VT origin. Our approach also correctly identified non-inducibility in patients without pathological wall-thinning (in this case using a cohort of patients with NICM without CCT evidence of wall-thinning).

In three patients with ICM, agreement was weaker, both with mapped circuits (patient 3) and VT origin surrogates (patients 5 and 8), who also showed few inducible VTs (Fig. 3). Despite an ischemic etiology, they exhibited minimal pathological LV wall-thinning on CCT, suggesting LVWT may be an inadequate scar surrogate in such cases. Direct LVWT analysis in these patients suggests that >10% of the LV with thinning <6 mm may be required for reliable model performance, though this guidance requires validation in larger cohorts.

The strength of correlations between simulated and clinical VT also determines model utility. In cases where the approach was less appropriate, few simulated VTs exceeded the correlation threshold for SATHe Map construction (Fig. 5), indicating weaker matches between simulated and clinical VTs. For example, patient 3 required lowering the threshold to 0.7 to generate any hotspot. Therefore, combining measures of LV wall-thinning with quantitative ECGs correlation may help identify patients for whom the approach is unsuitable, where the SATHe Map should not be used. This may be further refined by applying stricter correlation thresholds to ensure only well-matched circuits inform SATHe Map construction.

By quantifying confidence in model predictions, our approach provides a safeguard to identify when patient-specific models may be unreliable, representing an important strength in the clinical translation. In ischemic cases where wall-thinning does not accurately identify scar (for example patients 3, 5 and 8), applying the same framework to LGE intensity may improve targeting and extend applicability to non-ischemic cardiomyopathies.

### Clinically relevant guidance within an ablation workflow

A key contribution of this study is the introduction of a robust method to integrate multiple VT circuits from uncertainty analysis into a clinically actionable heat map (SATHe Map). The map is generated using a target VT ECG, available either pre-procedurally or acquired following induction. In the latter case, simulations can be pre-computed, with final ECG correlation performed in minutes during the procedure to produce the SATHe Map. The approach is based on a single VT template, but can be readily repeated for additional induced VTs to support comprehensive substrate targeting.

Notably, multiple simulated VT circuits (often from different model instances) showed similarly high correlations with the target ECG (Fig. 5), with at times visually different circuits, although sharing common isthmuses. Given this uncertainty, selecting a single ‘best’ circuit to guide ablation may be unreliable. Instead, by combining isthmus locations from all high-matching circuits, our approach more robustly identifies likely ablation targets despite scar uncertainty. This highlights the value of SATHe analysis as a complementary tool that can integrate with existing EAM systems to support real-time clinical decision-making.

### Future work

Future investigations will extend our framework to uncertainty in scar representation from high-resolution LGE-CMR within computational models, not possible in this cohort due to lack of patients with high-resolution wideband LGE-CMR. While LVWT is well established for identifying chronic scar in ICM (for guiding catheter ablation therapy^13,24^ and representation within image-based personalized models^8,9^), adapting our approach to LGE-based scars would facilitate application to patients who are non-ischemic, where LVWT is a less accurate surrogate of scars. This will require advanced wideband CMR techniques to enable high-fidelity imaging of patients with implanted devices. Incorporating uncertainty in electrophysiological parameters (such as scar/BZ conductivity, fibrosis representation and myocardial conduction velocity^25^) will also help assess the relative impact of structural versus functional uncertainty in predicting clinical VT circuits.

Further comprehensive clinical validation of the ablation targets identified by our approach, in either models reconstructed from CMR- or CT-based scar, will require detailed activation mapping of VT circuits in all patients within a larger cohort. Following comprehensive validation, full assessment of the utility of these methods can only be assessed in a prospective trial with ablations being guided by simulation output.

### Limitations

Practical ablation guidance with SATHe Map is limited by intra-procedural registration of the imaging-derived model with EAM geometry, a common challenge in image-guided ablation. Lower correlation values may arise from factors such as ECG electrode localization errors, simplified electrophysiological parameterization or inaccuracies in scar representation. Accordingly, simulated-clinical ECG agreement should be interpreted alongside wall-thinning maps, with SATHe Maps used as complementary, rather than standalone, guidance alongside imaging and EAM data. Incorporating spatial heterogeneity in tissue conductivity could further improve correlations and may be enabled by intra-procedural conduction velocity measurements from EAM.

## Methods

### Clinical data

The study included 14 patients undergoing clinically indicated catheter ablation for VT at St Thomas’ Hospital in the context of structural heart disease (11 ICM and 3 NICM). The study was conducted in accordance with the Declaration of Helsinki and was approved by the UK Health Research Authority Research Ethics Committee (ref. no. 22/LO/0907), with all patients providing written informed consent. All participants underwent a standardized workflow that included pre-procedural imaging and VT induction, allowing for comprehensive integration of anatomical and electrophysiological data in computational modeling.

#### Patient imaging and image processing

Before ablation, all patients underwent comprehensive ECG-gated cardiac CT imaging. In addition, fluoroscopy and post-procedure X-rays were obtained. An overview of how the different imaging modalities were processed and used to build the personalized computational models is provided below.

#### CT

Cardiac CT scans were acquired using a Siemens SOMATOM Force Dual Energy scanner operating at 100 kV, with fully retrospective ECG gating and intravenous contrast enhancement. The contrast agent (Omnipaque, GE Healthcare) was administered according to local clinical guidelines to ensure optimal vascular and myocardial opacification. These scans were primarily used to delineate cardiac anatomy, including the chambers, valves and major vessels. Additionally, a FLASH angiogram was performed to obtain full-body images of the patient’s torso, providing contextual anatomical detail for downstream modeling and electrode localization. From the acquired CT data, the optimal diastolic phase with maximal contrast enhancement was selected for segmentation.

The cardiac CT dataset was processed using NumeriCor’s Studio auto segmentation tool (https://numericor.at/rlb/wordpress/products/), which automatically identified key cardiac structures, including the LV myocardium, right ventricular blood pool, atria and major thoracic vessels. In some cases, minor manual refinements were required to correct for artifacts introduced by implanted cardiac devices.

#### Fluoroscopy/X-ray

Fluoroscopy performed during the procedure, along with post-procedure X-rays, was used to localize the ECG electrodes placed on the patients’ torsos. Radiopaque ECG electrodes enabled precise identification of their anatomical locations relative to internal cardiac landmarks.

#### Clinical ablation procedure and electro-anatomical mapping

Catheter ablation procedures were conducted in accordance with the institutional standard of care. Detailed EAM was performed in all patients using the CARTO 3.0 system, along with the use of intracardiac echocardiography (ICE). Radio-opaque ECG dots were applied along with CARTO navigation patches. Before anesthesia, VT was induced using a burst pacing protocol delivered via the patient’s indwelling device, with simultaneous recording of a full 12-lead ECG. VT was successfully induced in all patients with ICM. In the three patients with NICM, VT was non-inducible. ICE was employed to capture anatomical detail and assist with subsequent registration of EAM data to cardiac CT-derived models. ICE images were segmented to define surfaces for integration into the CARTO 3 EAM system using the CARTOSOUND module (Biosense-Webster). Substrate mapping was performed in sinus or paced rhythm and during VT at the operator’s discretion with a DECANAV (Biosense-Webster) multipolar mapping catheter and ablation delivered according to clinical standard of care via a 3.5-mm distal tip electrode SMARTTOUCH Surround-Flow bi-directional ablation catheter (Biosense-Webster). Following substrate mapping, pace mapping was performed using the ablation catheter and the PaSo module within CARTO 3. Ablation was delivered at the discretion of the operator and based on substrate mapping, pace mapping, activation and entrainment mapping where available.

Following the procedure, EAM data were exported and processed using Pyceps (v.1.0)^26^. The processed dataset included detailed voltage maps, anatomical geometry shells and spatial coordinates of all mapping points. Additional exports included ablation lesion locations and geometries derived from ICE imaging, such as the aortic cusps.

#### Clinical ECG processing

Twelve-lead ECGs recorded during VT induction were processed for use as clinical templates for comparison with the in silico VT. Segments showing successful monomorphic VT induction were identified and VT cycle length computed. Individual QRS complexes were extracted, with outlier beats excluded by comparing each beat to the median waveform, to produce an average QRS morphology representative of the induced VT.

### Computational model generation

An overview of the computational pipeline is illustrated in Fig. 1, highlighting the steps of biventricular (BiV) model construction, LVWT-based substrate characterization, and identification of relevant VT circuits.

#### Biventricular model generation

Segmented cardiac CT data were imported into NumeriCor’s CardioTwin BiV model generation pipeline (https://numericor.at/rlb/wordpress/products/), which automatically produced anatomically accurate, high-resolution finite element models of the BiV geometry (Fig. 8a). The resulting meshes had a spatial resolution of approximately 1,000 µm (isotropic).

Each model incorporated rule-based myocardial fiber orientation^27^. Universal Ventricular Coordinates (UVCs)^28^ were assigned to the mesh, providing a standardized coordinate system to facilitate inter-subject comparison. Non-conducting regions were defined around the mitral and tricuspid valve annuli to represent fibrous insulation.

#### Myocardial LVWT-driven substrate characterization with uncertainty quantification

LVWT was quantified directly from the CT-derived BiV models, defined as the distance between epicardial and endocardial mesh surfaces, shown in Extended Data Fig. 1, center. To achieve this, a distance field was computed between the endocardium, defined as the activation origin, and the epicardium as the termination surface^29^. Each finite element was assigned a value representing its endocardial distance. The process was then repeated to generate a distance field between the epicardium and endocardium, yielding a second set of values corresponding to epicardial distance. The sum of the two distances for each element provided a measure of local LV wall thickness within the model.

Regions of pathological LVWT were assessed by determining the relative percentage of LV < 6 mm. As LVWT is by definition a transmural field, only points on the LV endocardium were analyzed. To avoid physiologically thinned regions, the apex (UVC *z*-coordinate < 0.1) and valve-ring regions (*z*-coordinate > 0.8) were excluded.

To support substrate characterization, regions of myocardial thinning were identified based on previously established thresholds^8^ to represent both dense scar and BZ tissue within the computational BiV models. To assess the robustness of model predictions to variability in scar and BZ definitions, a threshold-sweeping approach was implemented. Multiple scar thresholds between 2 mm and 10 mm (in 0.5-mm increments) were applied across the LVWT maps. For each scar threshold, six BZ definitions were evaluated by incrementally extending the BZ upper bound by 0.5 mm to 3 mm relative to the scar threshold (scar + 0.5 mm to scar + 3 mm). For each combination of thresholds, the resulting tissue classifications were integrated into the BiV model (Extended Data Fig. 1, right) to create a subcohort of model ‘instances’. Subsequent simulations were conducted on all model instances to evaluate sensitivity of VT inducibility and isthmus localization to definitions of scar anatomy (Extended Data Fig. 1).

#### ECG electrode identification

Segmentation was performed automatically on whole-torso CT image datasets using the TotalSegmentator tool^30^ to extract noncardiac anatomical structures relevant for electrode localization and model integration. In addition to organ segmentation, CT-based intensity thresholding techniques were applied to identify and extract high-density objects such as implantable cardioverter-defibrillator leads and other metallic implants. The 3D isosurface meshes of the torso and internal structures, along with the internal metalwork, were then generated using Seg3D (https://www.sci.utah.edu/software/seg3d.html), allowing for accurate geometric representation of the thoracic anatomy and anatomical markers for spatial registration.

Anatomical models were co-registered with intra-procedural fluoroscopy and post-procedural X-rays to localize 12-lead ECG electrode positions relative to fixed landmarks on the patient torso (for example, implantable cardioverter-defibrillator leads and bony structures). For six earlier patients, corresponding X-ray and fluoroscopic images were not available. In these cases, standardized electrode placements were used, positioned on the patient-specific torso models.

### Identification and simulation of VT circuits within personalized BiV models

#### Electrophysiological modeling

Electrophysiological simulations were conducted using the BiV models incorporating substrate definitions based on LVWT within in each model instance, as previously described. A Reaction–Eikonal (RE) formulation was implemented via the CARPentry-Pro simulation platform (NumeriCor) to efficiently model electrical wavefront propagation while preserving activation accuracy^31^. This approach, implemented in the VITA tool^32^, enables rapid and automatic identification of all potential anatomical VT circuits within an image-based ventricular model in near-real time, along with simulation of corresponding ECG signatures of all identified circuits.

Myocardial conduction velocities were set to 0.63 m s^−1^ along the myofibre direction and 0.30 m s^−1^ in the transverse direction with reduced isotropic conduction velocity of 0.15 m s^−1^ assigned to BZ regions^19,21,22,33–36^. The scar tissue was modeled as non-conductive by applying a no-flux boundary condition at its interface effectively isolating the intracellular space within the scar. Cellular ionic dynamics were represented using the ten Tusscher human ventricular action potential model^37^. All simulation parameters, including solver settings and time step configurations, are provided in the Supplement to support reproducibility.

#### Virtual Induction and Treatment of Arrhythmias

VITA^21^ was applied to all patient model instances accounting for the uncertainty in scar substrate definition based on LVWT threshold, as described above, to identify all unique re-entrant circuit pathways exposed by the variations in scar architecture.

In brief, VITA applies a rapid RE model to sequentially pace the myocardium from multiple sites, identifying regions where wavefronts bifurcate and rejoin, which is indicative of protected isthmuses that may sustain re-entrant circuits. Potential re-entrant pathways are then probed by simulating unidirectional block at the isthmus exit or entrance, delineating the VT circuit and computing the time taken to complete a full circuit. Those VT circuits with round-trip times less than clinically relevant thresholds are discarded. For each remaining uVT circuit identified, both positive and negative chirality circuits were computed.

#### ECG VT simulation

For all uVT circuits uncovered, corresponding activation time maps were used in RE simulations to derive full transmembrane potential dynamics for a complete re-entrant cycle. Corresponding 12-lead ECGs were simulated from the transmembrane potential data by recovering extracellular potentials^38,39^ at each electrode position identified from the patient-specific torso anatomies, described above. The entire process of identifying all viable VT circuits for a single model instance, including ECG simulation and correlation analysis with the clinical template, consistently took less than 5 min on a desktop computer (24-core Intel Xeon Gold 6240 R CPU 4.0 GHz (64 bit Linux), 200 GB RAM).

### Comparison of simulated and clinical ECGs

#### Quantitative ECG comparison

In silico VT ECGs for each identified circuit were quantitatively compared to reference clinical ECGs. A dynamic temporal alignment approach was employed. Initially, simulated ECG signals were subjected to a pseudo functional conduction velocity personalization procedure, where each lead’s waveform was interpolated to simulate a range of temporal dilation or compression factors. The same factor was used across all leads each time.

This allowed for adjustment of the simulated ECG QRS duration to best align with the reference signals, effectively accounting for the unknown patient conduction properties. For each candidate stretch factor within a predefined range, the simulated ECG was then aligned with the clinical ECG using an energy-maximization technique to synchronize dominant waveform features. Following alignment, Pearson CCs (widely used in ECG analysis due to lack of dependence upon relative signal amplitude) were computed for each lead, with the top ten averaged to provide a robust measure of overall similarity for each stretch factor. The stretch factor yielding the highest average correlation was selected as optimal. The normalized signals from each lead after optimization were plotted together for visual assessment, with their CCs displayed, enabling detailed inspection of agreement across the 12 leads. The process of uncovering uVTs, simulating ECGs and aligning with clinical targets is shown in Extended Data Fig. 1 along with the central part of Fig. 1.

#### Identification of clinically relevant VT circuits via ECG correlation

All ECGs corresponding to unique VT circuits of both chiralities, identified across all patient-specific model instances reflecting uncertainty in LVWT-derived scar substrates, were correlated with the clinical target ECG trace to yield a single mean correlation score. These scores provided a quantitative basis for assessing the physiological relevance of each modeled VT circuit. The maximum correlation values were extracted to identify the simulation VT circuits best matching the clinical VTs.

### Construction of simulation prediction heat map for ablation target guidance

#### Automated identification of isthmuses from simulated VTs

Isthmus identification was performed by analyzing activation patterns from simulated VT episodes obtained through VITA. In VITA, circuits are simulated starting from sites of unidirectional block defined as exit or entrance sites to isolated isthmus regions. The initial period of tissue activation following this unidirectional block simulation was identified as the early onset of ventricular depolarization, estimated from simulated ECGs using vectorcardiographic (VCG) dipole analysis. Regions of tissue that activated before QRS take off (defined by reaching a normalized VCG dipole magnitude threshold of 0.05) were labeled as potential isthmus candidates. To account for spatial relevance, these binary isthmus regions were scaled based on their proximity to exit/entrance sites as identified by simulated LAT maps. Isthmus regions from positive and negative chiralities for each unique circuit were identified and combined to delineate full isthmus regions.

#### Combining spatial isthmus regions

All unique VT circuits which matched the target clinical VT ECG traces by exceeding a predefined CC threshold (>0.8) were taken forward to construct the ablation target guidance map. Note, for one patient (patient 3) a threshold of 0.7 was used, discussed in the text. Each identified isthmus from each qualifying circuit was then weighted by its correlation value (with respect to the clinical VT), interpolated onto a common anatomical reference mesh (the patient BiV model with no identified scar substrate), and summed across all model instances. The resulting composite map was normalized to produce a population-level heat map distribution highlighting the most consistent and clinically relevant isthmus locations across the dataset that matched the presenting clinical VT, accounting for uncertainty. This process is illustrated in the latter part of Fig. 1.

#### Comparison with clinical data

To enable direct qualitative comparison with clinical data, EAM geometries were registered to the patient-specific BiV models derived from cardiac CT. This process was performed manually using NumeriCor’s Studio software. Initial alignment was guided by the aortic cusps segmented from ICE data, which were matched to the aortic valve and ascending aorta in the BiV model. Registration was further refined using the LV and right ventricular endocardial shells from EAM, ensuring that these conformed closely to the corresponding surfaces in the computational model, acknowledging that, in some cases, the entirety of the LV wall may not have been fully mapped, leading to apparent misalignment. Nonetheless, the use of ultrasound fiducials to aid registration (in the same image space as the EAM data) ensures fidelity of the registration process even when an incomplete endocardial map is obtained.

All registrations were carried out by trained operators who were blinded to the VT induction data to avoid bias. Once complete, the transformation matrices were saved and applied uniformly to all exported EAM data. Clinically relevant points, including PaSo locations and ablation lesion points were projected directly onto the endocardial surface of the BiV model for later direct comparison with simulation data. Scar clinical data fields, such as bipolar voltage amplitude and VT LAT maps were visualized on the native CARTO shells, registered to the BiV models.

### Statistics and reproducibility

No statistical method was used to predetermine sample size and no data were excluded from analyses. All available patient data were used. The experiments were not randomized. Clinical investigators analyzing clinical data were blinded to simulation results.

### Reporting summary

Further information on research design is available in the Nature Portfolio Reporting Summary linked to this article.

## Supplementary information

Supplementary Information Full details of parameters required for simulated reproducibility using open source software.

Reporting Summary

Peer Review File

## Source data

Source Data Figs. 3 and 5. Raw data for all patients detailing all uVTs uncovered, along with CCs.

## Data Availability

The clinical data associated with this study cannot be made publicly available according to Guy’s & St Thomas’ NHS Foundation Trust’s policy. They can be accessed on an individual basis through a request to M.J.B. via an institutional data-sharing agreement. A subset of biventricular personalized models are uploaded as supplementary data, along with relevant metadata.

## Code availability

The algorithm underlying the VITA technology for finding VT circuits within a computational model is subject to filed patent. Correspondingly, full details of the code therefore cannot be shared; however, comparable induction protocols can be performed in OpenCARP (https://opencarp.org/), as described by Campos et al.^21^. Re-entrant VT circuits and ECGs were simulated using a proprietary version of CARPentry-Pro with an RE model. Equivalent simulations can be performed in OpenCARP using a reaction-diffusion formulation. Full simulation parameters are provided in Supplementary Information.
