# Supplementary material for: Accounting for uncertainty in computational models of ventricular tachycardia improves ablation guidance
Source: Nat Cardiovasc Res. 2026 Aug 14;5(9):807–18. doi: 10.1038/s44161-026-00856-w (PMC13561879; doi:10.1038/s44161-026-00856-w)
Supplement: Supplementary file 1 [file 44161_2026_856_MOESM1_ESM.pdf]

nature cardiovascular research

Supplementary information

https://doi.org/10.1038/s44161-026-00856-w

Accounting for uncertainty in computational models of ventricular tachycardia improves ablation guidance

In the format provided by the authors and unedited

Supplemental Information

The below tables specify the parameters required to reproduce simulations as used in this manuscript within the OpenCARP framework.

Cellular Electrophysiology

Table 1: Cellular electrophysiology (EP)

Parameter	Value	Description
-num_imp_regions	1	Number of regions with different EP properties
-imp_region[0].name	Myocardium	Name of the region
-imp_region[0].im	TT2	ten Tusscher-Panfilov 2006 model
-imp_region[0].cellSurfVolRatio	0.14	Surface-to-volume ratio

Conduction Properties

Table 2: Conduction properties
The below conductivity values represent tuned parameters, suitable to replicate the prescribed Reaction-Eikonal conduction velocities using in the manuscript, for meshes of resolutions ~1mm (as used in the manuscript) in the context of a reaction-diffusion monodomain representation in OpenCARP.

Parameter	Value	Description
-num_gregions	2	Number of regions with different tissue properties
-gregion[0].name	Myocardium	Name of the region
-gregion[0].num_IDs	N	Number of regions tagged as myocardium
-gregion[0].ID[0]	ID1	Tag of the first myocardial region
-gregion[0].ID[1]	ID2	Tag of the second myocardial region
-gregion[0].ID[N-1]	IDN	Tag of the N-th myocardial region
-gregion[0].g_il	0.261	Intracellular conductivity (IC) along the fibres (S/ m)
-gregion[0].g_el	0.9375	Extracellular conductivity (EC) along the fibres (S/ m)
-gregion[0].g_it	0.0285	IC transverse to myofibre direction (S/m)
-gregion[0].g_et	0.354	EC transverse to myofibre direction (S/m)
-gregion[1].name	BORDER-ZONE(BZ)	Name of the region
-gregion[1]. num_IDs	N	Number of regions tagged as BZ
-gregion[1].ID[0]	ID1	Tag of the first BZ region
-gregion[1].ID[1]	ID2	Tag of the second BZ region

-gregion[1].ID[N-1]	IDN	Tag of the N-th BZ region
-gregion[1].g_il	0.0285	IC along the fibres (S/m)
-gregion[1].g_el	0.354	EC along the fibres (S/m)
-gregion[1].g_it	0.0285	IC transverse to myofibre direction (S/m)
-gregion[1].g_et	0.354	EC transverse to myofibre direction (S/m)

Tissue regions tagged as scar are assumed to have [0 0 0] fiber direction, and were thus absent from the simulation domain.

Pacing Protocol

Table 3: Pacing protocol

In the manuscript, arrhythmia induction was conducted with our bespoke VITA approach (Campos et al, 2021). Pacing was carried out within the VITA framework from defined sites within the left ventricle using Universal Ventricular Coordinates (UVCs), ensuring standardised spatial localisation across patient-specific models. Each stimulus was applied at the designated UVC coordinates to probe for eligible VT circuits. As detailed in Campos et al (2021), replica induction experiments can be prescribed using a reaction-diffusion (monodomain) approach within OpenCARP using S1S2 protocols from the specified UVC locations below. Below, a single (S1) stimulus is shown. Additional stimuli (S2, S3) to facilitate arrythmia induction can also be included, with specific relative timings. Following induction, ECGs can be simulated using phie_recovery methods.

**Parameter**	**Value**	**Description**
-num_stim	1	Number of stimuli
-stimulus[0].stimtype	0	Type of stimulus (transmembrane current)
-stimulus[0].strength	100	Stimulus strength
-stimulus[0].start	0	Start time
-stimulus[0].vtx_file	StimFileName	File name with vertex-based electrode definition
-stimulus[0].duration	1	Duration of stimulus
UVC coordinates	[0.875, 0, 1.57, -1]; [0.875, 0, -0.52,-1]; [0.875, 0, -2.62, -1]; [0.875, 0, 2.62, -1]; [0, 0, 0, -1]	UVC coordinates where eligible VT circuits are identified from
-phie_recovery_file	Filename	Name of file to save recovered phie data to
-phie_rec_ptf	PtsFilename	Coordinates of locations to recover extracellular potential from

Table 4: Solver options

Parameter	Value	Description
-dt	25	Size of the time step
-parab_solve	1	Crank-Nicolson
-cg_tol_parab	1e-8	Solver tolerance
-operator_splitting	1	Use operator splitting
-mass_lumping	1	Lump mass matrix
