# Supplementary material for: Accounting for uncertainty in computational models of ventricular tachycardia improves ablation guidance
Source: Nat Cardiovasc Res. 2026 Aug 14;5(9):807–18. doi: 10.1038/s44161-026-00856-w (PMC13561879; doi:10.1038/s44161-026-00856-w)
Supplement: Supplementary file 2 [file 44161_2026_856_MOESM2_ESM.pdf]

nature portfolio

Corresponding author(s): Dr. Martin Bishop
Last updated by author(s): 01/07/2026

Reporting Summary

Nature Portfolio wishes to improve the reproducibility of the work that we publish. This form provides structure for consistency and transparency in reporting. For further information on Nature Portfolio policies, see our Editorial Policies and the Editorial Policy Checklist.

Statistics

For all statistical analyses, confirm that the following items are present in the figure legend, table legend, main text, or Methods section.

n/a	Confirmed	
☒	☐	The exact sample size (*n*) for each experimental group/condition, given as a discrete number and unit of measurement
☒	☐	A statement on whether measurements were taken from distinct samples or whether the same sample was measured repeatedly
☒	☐	The statistical test(s) used AND whether they are one- or two-sided<br>*Only common tests should be described solely by name; describe more complex techniques in the Methods section.*
☒	☐	A description of all covariates tested
☒	☐	A description of any assumptions or corrections, such as tests of normality and adjustment for multiple comparisons
☒	☐	A full description of the statistical parameters including central tendency (e.g. means) or other basic estimates (e.g. regression coefficient) AND variation (e.g. standard deviation) or associated estimates of uncertainty (e.g. confidence intervals)
☒	☐	For null hypothesis testing, the test statistic (e.g. *F*, *t*, *r*) with confidence intervals, effect sizes, degrees of freedom and *P* value noted<br>*Give P values as exact values whenever suitable.*
☒	☐	For Bayesian analysis, information on the choice of priors and Markov chain Monte Carlo settings
☒	☐	For hierarchical and complex designs, identification of the appropriate level for tests and full reporting of outcomes
☒	☐	Estimates of effect sizes (e.g. Cohen's *d*, Pearson's *r*), indicating how they were calculated

Our web collection on statistics for biologists contains articles on many of the points above.

Software and code

Policy information about availability of computer code

Data collection	Identification of VT circuits was conducted through the use of the Virtual Induction and Treatment of Arrhythmias, which is proprietary code subject to filed patent (EPEP21192067.3, publication n. EP4138093A1). Similar induction protocols can be performed with the OpenCARP [1] software (https://opencarp.org/) which is available to the community, as detailed in [2]. Simulation of subsequent reentrant VT circuits and corresponding ECGs was conducted with a proprietary version of the Cardiac Arrhythmia Research Package [3] (CARPentryPro) using the Reaction-Eikonal model [4]. These simulations can also be performed with a reaction-diffusion representation model with OpenCARP. Full parameter set for reproduction of these simulations is provided in the Supplement.<br><br>[1] Plank, G. et al: The openCARP simulation environment for cardiac elec- trophysiology. Computer methods and programs in biomedicine 208, 106223 (2021). https://doi.org/10.1016/j.cmpb.2021.106223<br>[2] Campos, F. O. et al. An automated near-real time computational method for induction and treatment of scar-related ventricular tachycardias. Med Image Anal 80, 102483 (2022).<br>[3] Vigmond, E. J. et al. Computational tools for modeling electrical activity in cardiac tissue. Journal of Electrocardiology 36, 69–74 (2003).<br>[4] Neic, A. et al. Efficient computation of electrograms and ECGs in human whole heart simulations using a reaction-eikonal model. Journal of computational physics 346, 191–211 (2017). https://doi.org/10.1016/ j.jcp.2017.06.020
Data analysis	No specific custom software beyond the above was used to analyse the data. Simulation results are presented directly as output from the software above.

For manuscripts utilizing custom algorithms or software that are central to the research but not yet described in published literature, software must be made available to editors and reviewers. We strongly encourage code deposition in a community repository (e.g. GitHub). See the Nature Portfolio guidelines for submitting code & software for further information.

Data

Policy information about availability of data

All manuscripts must include a data availability statement. This statement should provide the following information, where applicable:

- Accession codes, unique identifiers, or web links for publicly available datasets
- A description of any restrictions on data availability
- For clinical datasets or third party data, please ensure that the statement adheres to our policy

The clinical data associated with this study cannot be made publicly available according to Guy's & St Thomas' NHS Foundation Trust's policy. They can be accessed on an individual basis through a request to the corresponding author via an institutional data-sharing agreement: contact Dr Martin Bishop (martin.bishop@kcl.ac.uk) to facilitate this.
Requests should be made to the corresponding author who will aim to respond to such requests within 1 month.
A sub-set of bi-ventricular personalised models are uploaded as Supplemental Data, along with relevant meta-data.

Human research participants

Policy information about studies involving human research participants and Sex and Gender in Research.

Reporting on sex and gender	Sex and gender based analysis not carried out - patient cohort (ischemic cardiomyopathy with VT ablation) was small and hence would not have provided the depth of analysis for gender based differences in the results.
Population characteristics	Structural heart disease undergoing VT ablation
Recruitment	Fourteen patients undergoing clinically-indicated catheter ablation for VT at St. Thomas Hospital in the context of ischemic and non-ischemic cardiomyopathy. Collected clinical data was used retrospectively, and thus selection bias is not relevant.
Ethics oversight	UK Health Research Authority Research Ethics Committee (REC reference 22/LO/0907)

Note that full information on the approval of the study protocol must also be provided in the manuscript.

Field-specific reporting

Please select the one below that is the best fit for your research. If you are not sure, read the appropriate sections before making your selection.

☒ Life sciences ☐ Behavioural & social sciences ☐ Ecological, evolutionary & environmental sciences

For a reference copy of the document with all sections, see nature.com/documents/nr-reporting-summary-flat.pdf

Life sciences study design

All studies must disclose on these points even when the disclosure is negative.

Sample size	No sample size calculation was performed. We studied fourteen patients with ischemic cardiomyopathy that had undergone VT ablation. Sample size was determined by utilising all available patients and data.
Data exclusions	Patients that had not undergone VT ablation, under 18 years and pregnancy, contraindications to CT imaging or contrast administration, such as severe renal impairment or known allergy to iodinated contrast media.
Replication	Simulations and post-analysis were replicated and verified using the tools described earlier.
Randomization	There was no process of randomisation that came into this study (this is not a controlled randomised study)
Blinding	This study is a retrospective study, rather than a randomised controlled trial, hence no blinding was involved.

Reporting for specific materials, systems and methods

We require information from authors about some types of materials, experimental systems and methods used in many studies. Here, indicate whether each material, system or method listed is relevant to your study. If you are not sure if a list item applies to your research, read the appropriate section before selecting a response.

Materials & experimental systems

n/a	Involved in the study
☒	☐ Antibodies
☒	☐ Eukaryotic cell lines
☒	☐ Palaeontology and archaeology
☒	☐ Animals and other organisms
☐	☒ Clinical data
☒	☐ Dual use research of concern

Methods

n/a	Involved in the study
☒	☐ ChIP-seq
☒	☐ Flow cytometry
☒	☐ MRI-based neuroimaging

Clinical data

Policy information about clinical studies

All manuscripts should comply with the ICMJE guidelines for publication of clinical research and a completed CONSORT checklist must be included with all submissions.

Clinical trial registration	*Provide the trial registration number from ClinicalTrials.gov or an equivalent agency.*
Study protocol	*Note where the full trial protocol can be accessed OR if not available, explain why.*
Data collection	*Describe the settings and locales of data collection, noting the time periods of recruitment and data collection.*
Outcomes	*Describe how you pre-defined primary and secondary outcome measures and how you assessed these measures.*
