# Supplementary material for: Accounting for uncertainty in computational models of ventricular tachycardia improves ablation guidance
Source: Nat Cardiovasc Res. 2026 Aug 14;5(9):807–18. doi: 10.1038/s44161-026-00856-w (PMC13561879; doi:10.1038/s44161-026-00856-w)
Supplement: Supplementary file 3 [file 44161_2026_856_MOESM3_ESM.pdf]

nature portfolio

Peer Review File

Accounting for uncertainty in computational models of ventricular tachycardia improves ablation guidance

Corresponding Author: Dr Martin Bishop

This file contains all reviewer reports in order by version, followed by all author rebuttals in order by version.

Version 1:

Reviewer comments:

Reviewer #1

(Remarks to the Author)

This manuscript written by a group experienced in computational modeling, is of high significance as it describes a methodology of near real time methodology to identify potential VT circuits. Herein they propose that a patient digital twin model can be generated in 20 mins using Virtual Induction and Treatment of Arrhythmias (VITA) compared to 24-48 hrs of external high performance computing facilities which require reaction-diffusion approaches. This methodology has been previously published in Heart Rhythm and EP Europace, 2023 and 2024 by incorporating data from MRI. The application of this is ability to process uncertainty data, which is meant by acute clinical data, and able to risk stratification for patients at higher arrhythmic risk. This paper is to compare VITA with clinical data from VT patients.

1. Major weaknesses is the authors do not offer discussion as to what is the clinical uncertainty that needs to considered? That term is very vague in this context and not clear what the authors are referring to. It seems the only uncertainty is varying the thickness of scar or borderzone. If only using CT for tissue thickness then the model is limited in looking at one data type. Previously they have also looked at MRI scar, inflammation. Why is this not included in their analysis? In other words, the authors need to make a case that the model is good enough to predict clinically meaningful VT. Does the highest CC match the clinical VT? The model as they show in Results Figure 1, can be adjust for borderzone and scar but what is the threshold that should be used?

2. Figure 2: This Figure is very confusing. Are there some columns with 0 VTs? If so it should be made clear. How many columns have a value of ‘0’? How were the scar and border zone thresholds chosen? What were the peak CC for each patient? The authors should label the row Scar and Borderzone. It seems the value chosen for Scar and Borderzone is arbitrary. If different scar and borderzone thresholds uncover differ uVT in different patients then what should be used when modeling VT in patients and what is the clinical impact of this?

3. Figure 3: Why is the % mismatch between simulation and clinical ECG of VT so variable between patient 1, 2 and 3. What is the green checkmark likely VT for Patient 3? What is considered high fidelity, or acceptable CC?

4. SATHe maps why would the heat maps be in areas away from the activation path as in Patient 11? The true ablation sites show a large ablation burden suggestive of gross homogenization of the tissue, rather than focusing on one circuit. Does this mean there were multiple clinical VTs?

5. Figure 6. Patient 9 why is the shell registration show such difference from modelled shell to the mapped shell? How would that be used, implemented or rectified in a clinical workflow?

6. Please comment on causes for low CC in some patients. And why is it that CC of 0.75 in Patient 11 is sufficient to reproduce the clinical VT, although the tracings were not shown and merely described in the text.

7. Figure 7. Paitent 5 the SATHe Map is completely in a different location from the ablation points. Does this mean the ablation did not work or is it bc the lack of change in pathological thinning of the wall hinder the performance of the model?

Suggestions for improvement:

1. If the authors were to prove their model is of clinical impact they should study patients with no inducible VT on EPS and compare to inducible VT.

2. How much wall thinning is necessary for the model to work?

3. In the introduction the authors state “However, concerns regarding the practical implementation of these computational-demanding approaches, along with then robustness of simulation predictions with respect to uncertainty in clinical data used to reconstruct image-based models is preventing clinical translation.” What does this mean? Uncertainty in clinical data?

4. What is the importance of considering variations in reconstructed scar anatomy in order to extract the best-matching simulated circuit

5. What is a viable simulated circuits from all different model ‘instances’ into a simulated ablation.

(Remarks on code availability)

Reviewer #2

(Remarks to the Author)
The authors should consider whether their models are digital twins, or personalised models. Most definitions of a digital twin particularly in traditional engineering settings include a dynamic updating of the twin each time new data from its physical counterpart become available, so that the twin is a detailed copy of the physical artifact.

I would take issue with the sentence in the introduction "Furthermore, a model’s ability to identify all potential circuits may more comprehensively identify relevant targets ...". This is a strong statement, and I would prefer to see a more nuanced form of words that acknowledges that models are not only intrinsically limited (they are models, not the patient) but also constrained by the information that is used to calibrate them. I agree that model-based approach can provide comprehensive information about possible re-entry circuits more quickly than a clinical investigation, but I don't agree that a model will always identify all potential re-entry circuits.

The tension between simulations and reality also extends somewhat into the rest of the manuscript, where it is not always easy to quickly distinguish simulated and actual re-entry circuits. For example, in Figure 3 it is not immediately clear if the LAT maps are actual VTs from clinical mapping, or simulated. I would recommend introducing some clear terminology both in the text as well as in figure legends and labels, that clearly identifies simulated and real results and observations.

The structure of the manuscript makes for hard reading because there are methodological details that are not clearly explained in the results section. For example the peak correlation coefficient (CC) should be defined in the main body of the results because this information is is needed to interpret Figure 2 and 4. An overall workflow figure (possibly an expanded form of Figure 9) in the earlier part of the manuscript that illustrates the processing pipeline would be of great help for the reader, and would enable a more effective explanation of Figure 4

While the overall modelling approach is sound and reasonable, I wonder if a fixed conduction velocity may be a problem. In principle, variations in conduction velocity, especially in scar border zones, could affect VT wavelength and period, which in turn could affect the viability of each isthmus and complicate calibration agains ECG signatures of VT. I do not suggest calibrating to conduction velocity, but I think this should be discussed as a potential limitation.

Minor points:

In places the use of words makes for slightly awkward reading, for example 'computational-demanding approaches' in the abstract. It might be helpful for a non-expert to read through the manuscript to check for readability.

Figure 1 would have greater impact if the simulated VT circuits could be illustrated for each of the three thresholds.

Figure 2 has been squashed along the horizontal axis.

It would be helpful to include some information about the computational resources required to run the simulations, and in particular whether the workflow could be completed in a timescale that could be fast enough for the clinical setting.

(Remarks on code availability)
OpenCarp is a software tool, which can be downloaded and installed. It is well recognised in the community, and widely used. While the simulations described in the manuscript are in principle reproducible, I could not find links to any of the anatomical models or specific metadata to run the simulations.

Version 2:

Reviewer comments:

Reviewer #1

(Remarks to the Author)
This reviewer appreciates the modification to the paper which has made it stronger however the revised paper still has some weaknesses that can be improved upon.

Figure 4. Shows Patient 8 has CC of 0.84 and patient 9 has CC of 0.83, however patient 8 has lack of concordance in leads V4, V5 and V6 whereas the text says Patient 9 only has lack of agreement on lead V4. These values does not seem to make sense. How can this be?

Figure 6. What weakens this figure is the lack of coregistration between the SATHE maps and what is maps on the bipolar map or VT LAT Map.

In Patient 11 the area highlighted on the SATHe map does not correlate with the area on tbe bipolar and VT LAT map. What is the purpose of the maps with the ablation lesions? It does not seem the ablation lesions correlate what was mapped with the bipolar maps. If the ablation lesions were able to terminate the VT then why where they placed there and not where the SATHe Map suggested?

Figure 8 why is the shell registration in patient 5&8 poorly co-registered?

(Remarks on code availability)

Reviewer #2

(Remarks to the Author)
The revised manuscript is a much easier read. In particular Figure 1 is a great improvement, and makes the overall workflow much clearer to understand.

All of my comments on the initial draft have been addressed to my satisfaction.

(Remarks on code availability)

Version 3:

Reviewer comments:

Reviewer #1

(Remarks to the Author)
I have no further comments. I believe that the authors have made modifications in the manuscript to address their limitations and have explained in depth the challenges to coregistration

(Remarks on code availability)

Open Access This Peer Review File is licensed under a Creative Commons Attribution 4.0 International License, which permits use, sharing, adaptation, distribution and reproduction in any medium or format, as long as you give appropriate credit to the original author(s) and the source, provide a link to the Creative Commons license, and indicate if changes were made.
In cases where reviewers are anonymous, credit should be given to 'Anonymous Referee' and the source.
The images or other third party material in this Peer Review File are included in the article's Creative Commons license, unless indicated otherwise in a credit line to the material. If material is not included in the article's Creative Commons license and your intended use is not permitted by statutory regulation or exceeds the permitted use, you will need to obtain permission directly from the copyright holder.
To view a copy of this license, visit https://creativecommons.org/licenses/by/4.0/

Editor's Feedback

We thank the Editor for their own appraisal of our work and for providing a detailed list of concerns to be prioritised. In addition to the detailed responses to individual specific Reviewer comments below, we have also briefly summarised how we have addressed these key issues identified by the Editor.

1) Substantially rewrite the manuscript to make it more accessible and provide additional methodological details (both referees).

We have thoroughly revised all aspects of the text, particularly the Introduction and Discussion, with a specific goal of enhancing clarity and accessibility. A consequence of this re-write was also to shorten and focus many aspects, providing more text space to thoroughly address the Reviewer concerns highlighted below. We have also significantly improved the figure captions and made additions to the Methods section, facilitating reproducibility of our work - see specific Reviewer comments for more details.

2) Include an analysis of patients without inducible VT and comment on the extent of wall thinning necessary for the model to function, as recommended by referee #1.

To address this point, we have added additional clinical data from three new patients who were non-inducible for VT. This allowed us to demonstrate that our modelling approach successfully identifies non-inducible patients, as our VITA algorithm also does not find any viable VT circuits in these patients, as expected. This is now made clear in the manuscript. We have also used this new analysis, alongside additional separate analysis of relative wall-thinning in all patients, to include additional guidance related to the extent of wall thinning necessary for model execution, as suggested. We believe this additional work (increasing our cohort to 14 patients) has improved the robustness of our study. See Suggestions 1 and 2 from Reviewer 1.

3) Clarify the importance of considering variations in reconstructed scar anatomy to identify the most accurate simulated circuit and how to identify viable circuits that can lead to successful ablations, as requested by referee #1.

This issue related to how variations in reconstructed scar anatomy help identify the most accurate simulated circuit is critical to our study. We have taken great care in now clarifying this important issue in the text. We have also included a more detailed description of how viable circuits can be identified from our work, alongside an additional figure (new Figure 2 & Figure 3B) more clearly highlighting how variations in scar anatomy influence optimal circuit detection. See Comment 1 and Suggestion 4 from Reviewer 1.

4) Provide links to the anatomical models and specific metadata to run the simulations, as requested by referee #2 in their remarks on code availability.

We entirely agree with the importance of reproducibility and appreciate that providing the anatomical models themselves represent an important aspect of facilitating this. In order to facilitate maximal reproducibility and promote transparency we have now uploaded as Supplemental Data two bi-ventricular anatomical models for representative patients. These models include myofibre orientations, universal coordinates and relevant tissue tags, as well as the essential left-ventricular wall thickness data files which can be used to defined scar/ borderzone (at varying thresholds) as implemented in this work. See comment by Reviewer 2.

Reviewer 1

This manuscript written by a group experienced in computational modeling, is of high significance as it describes a methodology of near real time methodology to identify potential VT circuits. Herein they propose that a patient digital twin model can be generated in 20 mins using Virtual Induction and Treatment of Arrhythmias (VITA) compared to 24-48 hrs of external high performance computing facilities which require reaction-diffusion approaches. This methodology has been previously published in Heart Rhythm and EP Europace, 2023 and 2024 by incorporating data from MRI. The application of this is ability to process uncertainty data, which is meant by acute clinical data, and able to risk stratification for patients at higher arrhythmic risk. This paper is to compare VITA with clinical data from VT patients.

We thank the Reviewer for their positive assessment of the overall significance of our work.

1. Major weaknesses is the authors do not offer discussion as to what is the clinical uncertainty that needs to considered? That term is very vague in this context and not clear what the authors are referring to.

We regret that the Reviewer felt we had not adequately clarified the clinical uncertainty our study aims to assess. We have thoroughly revised numerous areas of the manuscript where such clinical uncertainty is referred, clarifying which aspects are important and how they are addressed in this specific work. [See also response to Reviewer's Suggestion 3.]

- See Abstract: "*given uncertainty in …* "
- Introduction para 3: "*This introduces significant …"*
- Discussion (Importance of Considering Uncertainty): numerous clarifications throughout.

It seems the only uncertainty is varying the thickness of scar or borderzone. If only using CT for tissue thickness then the model is limited in looking at one data type. Previously they have also looked at MRI scar, inflammation. Why is this not included in their analysis?

The Reviewer raises an important issue which we agree was not sufficiently addressed in the manuscript. In fact, the purpose of this work was to present a novel methodological approach for incorporating uncertainty in personalised image-based models used to simulate reentrant VT circuits for ablation guidance. Our specific focus here is to assess the clinical uncertainty in the definition of scar substrate defined through CT-based wall thickness. This has been clarified throughout.

- See Introduction, para 6: "*in clinical imaging …* " and throughout
- See Results (Implication of Uncertainty in Scar Threshold and VT Inducibility): "*derived from CT …* "
- Discussion, para 1: "*in CT-derived scar …* "

The approach, however, is generic, and could indeed by applied to scar defined through cardiac Magnetic Resonance (CMR) imaging, for example, through varying threshold limits applied to contrast-enhanced images. Unfortunately, our currently-available patient cohort did not contain sufficient patients with adequate CMR data to investigate this; hence, our choice to focus on CT-based scar reconstruction, which is a very commonly used approach in chronic ischemic patients (pioneered by the Bordeaux team), currently the focus of a number of clinical studies and trials [PMID: 41667137]. We have clarified the specific focus of this work on CT-based scar uncertainty throughout, along with the widespread use of CT in defining scar and guiding ablation. We have furthermore explicitly referenced the adaptability of this approach to LGE-CMR based models, and included explanation of why this was not possible in this current cohort.

- Discussion (Future Work): "*not possible in …* "

In other words, the authors need to make a case that the model is good enough to predict clinically meaningful VT.

This is a really important issue and we apologise this was not made sufficiently clear previously. Acknowledging the importance of this point, we have worked hard to address this in our revised manuscript. The utility of CT-based scar definition has been addressed above. Furthermore we have now emphasised the close comparison of the simulated ECG traces with the clinical ECGs in Figure 4 (old 3), along with the matching of ablation targets with the SATHe Maps. We believe these data robustly demonstrate the ability of our presented method to predict the patient's clinical VT. We also further re-iterate that, to date, no other simulation study attempting to simulate patient VT circuits have ever presented ECG-based comparisons as we have done here, which we therefore believe represents a novel and very important characteristic of our data. We have now clarified this in the text.

❖ See Discussion (Validation of VT Circuit ECG Signatures): "*something not attempted … "* and "*Nevertheless, the consistently*…"

Does the highest CC match the clinical VT? The model as they show in Results Figure 1, can be adjust for borderzone and scar but what is the threshold that should be used?

The Reviewer raises a very important issue that we fully agree needs further clarification in the text. As shown in Figure 4 (old 3), the simulated VT with the highest matching CC indeed represents a very close match to the ECG signature of the clinical VT. However, as the Reviewer rightly notes, slight modifications in the scar/borderzone anatomy through variations in respective thresholds can yield important variations in the pro-arrhythmic substrate that nonetheless gives rise to simulated ECG signatures with very similar CC when compared to the clinical VT (shown in Figure 3 (old 2)). This is exactly the important variation and uncertainty we are seeking to address with our SATHe Map approach. By combining critical anatomical information from multiple simulated VT circuits, obtained through threshold variations, our method can provide clinicians with a single heat-map target. We have now included further clarity in this important issue in the text.

❖ See Discussion (Clinically-Relevant Guidance within an Ablation Workflow), para 2: "*Given this uncertainty … "*

Regarding which exact threshold to use, this is an extremely important point which we are pleased to clarify. Due to the high variation in the matches between simulated circuits with the target clinical traces (highlighted so clearly in Figure 3 (old 2) as the specific threshold of scar/borderzone is varied), it is not possible, or recommended, to define an exact 'one-size-fits-all' threshold. As implemented in this work, our approach specifically sweeps over a range of thresholds defining scar and borderzone, made possible due to the rapid nature of our simulation technology, searching for the simulated VT that best matches the clinical VT. This is a central conclusion of our study which we have taken the effort to clarify in the revised text.

❖ See Discussion (Importance of Considering Uncertainty): "*The strong sensitivity … "*

2. Figure 2: This Figure is very confusing. Are there some columns with 0 VTs? If so it should be made clear. How many columns have a value of '0'? How were the scar and border zone thresholds chosen? What were the peak CC for each patient? The authors should label the row Scar and Borderzone. It seems the value chosen for Scar and Borderzone is arbitrary. If different scar and borderzone thresholds uncover differ uVT in different patients then what should be used when modeling VT in patients and what is the clinical impact of this?

We apologise to the Reviewer for the lack of clarify in Figure 3 (old 2) and have made significant efforts to improve the figure itself, and the associated explanations. Briefly:

- There were indeed many cases of model instances that yielded 0 VTs. This has now been clarified in the caption.
- The peak CC has now been added as an annotation (replacing the 'star').
- Labels for the rows Scar and Borderzone have been added.

- For all patients, scar thresholds from 2mm-10mm (1mm increments) were swept over with corresponding alterations in borderzone in 0.5mm increments either side. This has been clarified in the Methods. In the figure itself, only data for 3 scar increments/thresholds either size of the threshold yielding the max CC are shown for clarity. This has now been clarified in the caption (and main text).
- Clarification of the thresholds used to define the model instances in the Methods.
- The issue related to patient-specific thresholds relates to Comment 1, above, which has been clarified in the Discussion.
- We also note that we have added a complementary figure panel in response to the Reviewer's later comment, more explicitly showing max CC values for each model instance.

❖ See Figure 3 (old 2) & caption.
❖ See additional Figure 3 (old 2) panel B.
❖ See Methods (Myocardial LVWT-Driven Substrate Characterisation with Uncertainty Quantification), para 2: "*Multiple scar thresholds … *"
❖ See Discussion (Importance of Considering Uncertainty): "*The strong sensitivity … *"

3. Figure 3: Why is the % mismatch between simulation and clinical ECG of VT so variable between patient 1, 2 and 3. What is the green checkmark likely VT for Patient 3? What is considered high fidelity, or acceptable CC?

All patients presented in Figure 4 (old 3) show close matches between simulated VTs and clinical VTs at >80%. Given the simplified nature of a computational model, we acknowledge that 'perfect' matches are unlikely. We therefore submit that these chosen patients all represent close matches with the clinical VT and have clarified this in the text.

❖ Refer to existing Discussion (Validation of VT Circuit ECG Signatures), para 2: "*Given the inherent … *"

Regarding the green checkmark, this was merely to demarcate a 'good' match and has simply been removed to avoid confusion.

Regarding the acceptable CC, this is addressed in the Methods section in the creation of the SATHe Maps. Here, only simulated VTs that provided matches with the clinical VTs >0.8 were taken forward for SATHe Map construction. The resulting SATHe Maps showed close association with mapped VT circuits (Figure 6 (old 5)) and surrogates of VT site-of-origin (Figure 7 (old 6)). Reducing this acceptable CC threshold resulted in SATHe Maps which were less strongly associated with the clinical VT maps. This important issue is now clarified in the Discussion.

❖ See Discussion (Validation of VT Circuit ECG Signatures), para 2: "*Nevertheless, the consistently … *"

4. SATHe maps why would the heat maps be in areas away from the activation path as in Patient 11? The true ablation sites show a large ablation burden suggestive of gross homogenization of the tissue, rather than focusing on one circuit. Does this mean there were multiple clinical VTs?

Our interpretation of Figure 6 (old 5) (Patient 11) is that the SATHe Map in fact aligns very closely indeed with the activation path. A careful explanation of these close matches, for Patient 11 and the other two patients in whom VT mapping was undertaken, was already in the text.

❖ See Results (*Comparison of SATHe Map Predictions with Clinical Measurements*): "*Across the three … *"

The Reviewer correctly identifies that an extensive region of ablation was undertaken in this patient, in keeping with the chosen ablation approach which targeted comprehensive substrate elimination. The SATHe Map reconstruction incorporates a single target clinical ECG only. The SATHe Map shown in Figure 6 (old 5) for Patient 11 was reconstructed based on the VT ECG associated with the induced and mapped VT, also shown in the activation map. Had additional clinical VT ECG traces been available for this patient, additional SATHe Maps may have been

constructed to potentially identify additional areas to target. We are grateful to the Reviewer for raising this important point, and have now fully clarified this in the text.

❖ See Discussion (Clinically-Relevant Guidance within an Ablation Workflow), para 1: "*The approach is … *"

5. Figure 6. Patient 9 why is the shell registration show such difference from modelled shell to the mapped shell? How would that be used, implemented or rectified in a clinical workflow?

The Reviewer is correct to seek clarity on this important issue. Although the surfaces match closely between the EAM shell and model endocardial surface in the mid-regions, it is true that extent of the EAM geometry does not extend quite as far in the basal or apical regions as the 'true' endocardium, represented by the CT-based model. In this case, the mapping catheter was not advanced into these areas during the procedure to accurately capture the full endocardial surface. This issue has been mentioned now in the Methods.

This does however permit us to highlight a strength in the methodology applied here. While many approaches to registration depend on a comprehensive endocardial geometry being acquired, we have employed ultrasound derived fiducials in the same image space as the EAM data to register the EAM data with the bi-ventricular models. This means that the fidelity of the registration process is intact even when an incomplete endocardial map is obtained. A complete endocardial geometry is not always possible to acquire for a range of procedural reasons, and importantly it may not always be appreciated that this is the case during a procedure, or indeed where the unmapped portion of the ventricle is located. The considerations highlight the value of a registration process that is independent of the acquired LV EAM geometry, and therefore we believe the technique applied here is the most robust approach to computational model registration that is available at present.

❖ See Methods (Comparison with Clinical Data): "*acknowledging that, in … *"

How this should be implemented/rectified in a clinical workflow relates to an issue that affects all types of image integration into EAM systems for ablation guidance (not just image-derived models). As noted above we would argue that the most defensible registration process is with the benefit of integrated real-time ultrasound imaging (i.e. intracardiac echocardiography) within an EAM. This is currently only available in Carto but is expected soon in Ensite as well. A range of the approaches to image registration have been taken and reported and all are subject to different challenges and sources of error. Also note that the SATHe map could be used in isolation, where the guidance could be displayed independently of the EAM on the personalised BiV shell model in which case it would provide the operator with an anatomic references for guidance. We have now included a discussion of this issue in the Limitations section.

❖ See Discussion (Limitations): "*Practical ablation guidance … *"

6. Please comment on causes for low CC in some patients. And why is it that CC of 0.75 in Patient 11 is sufficient to reproduce the clinical VT, although the tracings were not shown and merely described in the text.

We appreciate the Reviewer's request for clarity on this important issue. As mentioned above, there are a number of reasons why a VT simulated in a model may not produce a CC which matches exceptionally well with the clinical target VT, even if the 'correct' circuit is produced. These important issues are further clarified in the Limitations. Ultimately, the numerical value of the CC (and resulting SATHe Maps) should be viewed in the context of the pathological wall-thinning maps. This issue is also discussed in the context of the Reviewer's later suggestion.

❖ See Discussion (Limitations): "*Lower correlation values …*"

7. Figure 7. Paitent 5 the SATHe Map is completely in a different location from the ablation points. Does this mean the ablation did not work or is it bc the lack of change in pathological thinning of the wall hinder the performance of the model?

This Reviewer is entirely correct here. In all patients in Figure 8 (old 7), a lack of pathological wall-thinning results in a relatively small number of VTs being induced in these patients (seen in Figure 3 (old 2)), the majority of which correlate relatively poorly with the clinical VT (Figure 5 (old 4)). Following the Reviewer's suggestion below, we have also now included specific guidance as to the levels of wall-thinning required for model execution.

❖ See Discussion (Comparison with Clinical VT EAM Data): "*who also showed …* " and "*Direct analysis of …* "

Suggestions for improvement:

1. If the authors were to prove their model is of clinical impact they should study patients with no inducible VT on EPS and compare to inducible VT.

We entirely agree with the need to enhance the clinical impact of our work. In-light of this suggestion, we have included an additional 3 patient datasets, corresponding to patients who were non-inducible for VT upon programmed electrical stimulation. Reconstruction of patient-specific models from these patient datasets, and application of our VITA algorithm, indeed resulted in no VT circuits been located in any of these patients. This is entirely explained by analysis of the LV wall thickness in these patients, which demonstrated no pathological wall-thinning at all. This additional result is now included in the main text, along with the calculations of the wall-thinning in these non-inducible patients and relevant discussion.

❖ See Results: Utility of Model in Predicting Non-Inducibility
❖ See Methods: Clinical Data & Clinical Ablation Procedure
❖ See Discussion: Comparison with Clinical VT EAM Data: "*Our approach also …*"

2. How much wall thinning is necessary for the model to work?

This is a very important question raised by the Reviewer, but also one that is difficult to directly answer. In brief, it is not necessarily just the absolute value of the wall-thinning, but also its distribution within the LV, particularly with respect to channels of thicker tissue through chronically-thinned areas that may provide viable isthmuses forming circuits to sustain VT. Although studies suggest dense scar is associated with highly thinned ventricle wall (~<3mm), our results emphasise the importance of sweeping over a larger range of thresholds in order to uncover the best matching VT circuit. This is imperative due to the potential clinical uncertainty in imaging the LV and importantly its accurate segmentation and defining/computing LV wall thickness, due to the number of differing specific techniques used.

In-light of the Reviewer's suggestion, we have now included additional specific analysis of relative wall-thinning in patients to provide more direct guidance on model utility. In all patients in whom the model was deemed to have not provided useful guidance (specifically all three patients in Figure 8 (old 7), plus additional Patient 6 in whom ablation was not performed), LV wall thinning <6mm was present in <10% of their LV (mean 5.4%, min 3.5%, max 7.4%). In contrast, all patients in whom the model provided guidance that matched closely with ablation targets (Figures 6 & 7 (old 5 & 6)) consistently had >10% LV with wall thinning <6mm (mean 19.6%, min 11.7%, max 27.1%). We have now incorporated this additional analysis and guidance directly into the manuscript.

We also importantly note that it is not necessarily just the level of wall-thickness (specifically the threshold used to define dense scar), but also the numerical match of the CC (for that model threshold) with the clinical ECG. In-depth discussion of this important issue, including the highlighting of this new guidance for the practical use of our approach, is now provided in the discussion.

❖ See Methods (Myocardial LVWT-Driven Substrate Characterisation with Uncertainty Quantification): "*Regions of pathological …"*

- ❖ See Results (Comparison of SATHe Map Predictions with Clinical Measurements): "*Specifically, LV walling-thinning…* "
- ❖ See Discussion (Comparison with Clinical VT EAM Data): "*Direct LVWT analysis …* "

3. In the introduction the authors state "However, concerns regarding the practical implementation of these computational-demanding approaches, along with then robustness of simulation predictions with respect to uncertainty in clinical data used to reconstruct image-based models is preventing clinical translation." What does this mean? Uncertainty in clinical data?

We apologise for the slightly ambiguous nature of this statement. Here, we were specifically referring to uncertainty in the analysis, processing and interpretation of the clinical imaging data used to reconstruct the patient-specific anatomical models, in particular the scar imaging data. For example, uncertainty in which scar threshold to use in order to segment and define scar (either from CT-based wall thickness, as in this work, or from contrast-enhanced LGE CMR data) can have dramatic effects on the induced VT (as we categorically demonstrate in Figures 2-4 (old 1-3)). This issue is particularly problematic in the studies referred to here because they are so computationally-demanding it is simply not possible to consider more than a single model instance, and so cannot account for or quantify this uncertainty.

These important issues are further clarified now in the text (in the Abstract - where the above sentence was originally), but also in the Introduction.

- ❖ See Abstract: "*given uncertainty in …* "
- ❖ Introduction para 3: "*This introduces significant …*"

4. What is the importance of considering variations in reconstructed scar anatomy in order to extract the best-matching simulated circuit

We apologise that this key element of our study was not more clearly represented in the text. The simple answer here is "imperative". This should be emphasised most clearly in Figure 3 (old 2). Here, we see that, as scar anatomy is altered (through threshold changes used to define it) the number of VTs detected by the model can vary substantially.

Regarding how the closeness of the match varies, we have taken onboard the Reviewer's request and have created a new, additional figure. Here, we plot how, for different scar thresholds, the maximum CC of all of the uncovered VTs varies. As seen, for very small variations in scar thresholds (+/- 0.5mm) there are large changes in the match of the VT with the clinical trace. This truly underscores the importance of accounting for uncertainty in scar definition in order to uncover the best matching VT circuit.

- ❖ See Results, Figure 3B.

We have also added specific clarification of this key point to the Discussion.

- ❖ See Discussion (Importance of Considering Uncertainty), para 3: "*The strong sensitivity …* "

5. What is a viable simulated circuits from all different model 'instances' into a simulated ablation.

We interpret this suggestion as querying how simulated circuits from all model instances could be combined into a predicted ablation target. As detailed in the manuscript, ECGs corresponding to all simulated circuits uncovered by the model are correlated with the clinical ECG target. Those which are highly correlated (>0.8) are then used to create the SATHe Map. This involves identifying the critical part of the associated VT circuit - defined as isthmus tissue either side of the exit site (determined by analysis of the take-off of the QRS following pacing at this site). All critical regions associated with all highly correlated VTs are then combined (spatially) to produce the heat map. This is plotted on the default model (i.e. no scar), scaled 0-1 to identify target 'hot-spots'.

We apologise if this description was not sufficiently clear before. We believe that this methodology was described comprehensively in the Methods, but certainly appreciate that this could have been sign-posted forward better in the Results text, which we have now done.

❖ See Results (Comparison of SATHe Map Predictions with Clinical Measurements), para 1.

Reviewer #2

The authors should consider whether their models are digital twins, or personalised models. Most definitions of a digital twin particularly in traditional engineering settings include a dynamic updating of the twin each time new data from its physical counterpart become available, so that the twin is a detailed copy of the physical artifact.

We entirely agree with the Reviewer on this important issue. We had initially chosen to use this specific term in this context to be in-line with the numerous recent studies which directly refer to their personalised anatomical models (with no updating, etc) as 'cardiac digital twins'. In-light of this suggestion, we have removed this terminology throughout. Note, we kept this term in the first sentence of the abstract as it was in quotations, and was referring more to the overall field, rather than this particular work, which we feel is important to draw the attention of a more general audience to our work.

I would take issue with the sentence in the introduction "Furthermore, a model's ability to identify all potential circuits may more comprehensively identify relevant targets ...". This is a strong statement, and I would prefer to see a more nuanced form of words that acknowledges that models are not only intrinsically limited (they are models, not the patient) but also constrained by the information that is used to calibrate them. I agree that model-based approach can provide comprehensive information about possible re-entry circuits more quickly than a clinical investigation, but I don't agree that a model will always identify all potential re-entry circuits.

We thank the Reviewer for raising this important issue. We have now indeed toned-down the nature of this specific statement, as requested. We have also included discussion on the topic of the fidelity of the match between model simulations and patient data in the Discussion, related to comments above from Reviewer 1.

- ❖ See Introduction, para 2: "*Importantly, models can …* "
- ❖ See Discussion (Validation of VT Circuit ECG Signatures): multiple changes

The tension between simulations and reality also extends somewhat into the rest of the manuscript, where it is not always easy to quickly distinguish simulated and actual re-entry circuits. For example, in Figure 3 it is not immediately clear if the LAT maps are actual VTs from clinical mapping, or simulated. I would recommend introducing some clear terminology both in the text as well as in figure legends and labels, that clearly identifies simulated and real results and observations.

We have taken this important comment onboard and clarified in the text throughout the presentation of simulated vs clinical data, particularly figure captions.

- ❖ See Figure 4 (old 3), caption.
- ❖ See throughout the text.

The structure of the manuscript makes for hard reading because there are methodological details that are not clearly explained in the results section. For example the peak correlation coefficient (CC) should be defined in the main body of the results because this information is is needed to interpret Figure 2 and 4. An overall workflow figure (possibly an expanded form of Figure 9) in the earlier part of the manuscript that illustrates the processing pipeline would be of great help for the reader, and would enable a more effective explanation of Figure 4

We agree with the Reviewer regarding the issue of having the Methods separately at the end of the text. We have now revised much of the Results in order to explain important terms to assist in the interpretation of Figures, as well as adding more detail to figure captions. We have also more clearly sign-posted forward to key aspects of the Methods to assist in understanding.

- ❖ See Results, significant changes throughout text.

Furthermore, we have also actioned the request of the Reviewer to move Figure 9 further forward. We have now included this at the start of the Results, along with a short explanation and expanded caption. We agree that this has substantially helped the readability of the manuscript and in particular interpretation of the initial figures that immediately follow.

- ❖ See Results, para 1.
- ❖ See (new) Figure 1.

While the overall modelling approach is sound and reasonable, I wonder if a fixed conduction velocity may be a problem. In principle, variations in conduction velocity, especially in scar border zones, could affect VT wavelength and period, which in turn could affect the viability of each isthmus and complicate calibration agains ECG signatures of VT. I do not suggest calibrating to conduction velocity, but I think this should be discussed as a potential limitation.

The Reviewer raises a very important point. In fact, conduction velocity was modulated in borderzone areas, relative to healthy myocardium. In relation to assigning further conduction heterogeneity (i.e. full spatial heterogeneity, not just two regions of borderzone and healthy), we agree that this might be feasible in the future. However, it would also likely require intra-procedural updating of the model using detailed catheter-based electrogram measurements to compute and assign regional conduction (which itself, may also be prone to error), adding significant complexity to our approach and rendering it no-longer a pre-procedural planning tool. Nonetheless, this is a very important point, which we are pleased to now include a discussion of in the Limitations.

- ❖ See Discussion, Limitations: "*Incorporating spatial heterogeneity …* "

Minor points:

In places the use of words makes for slightly awkward reading, for example 'computational-demanding approaches' in the abstract. It might be helpful for a non-expert to read through the manuscript to check for readability.

We have thoroughly gone through all text and improved readability, involving significant re-writes to the majority of the Introduction and Discussion.

Figure 1 would have greater impact if the simulated VT circuits could be illustrated for each of the three thresholds.

We have actioned this request and have now added all simulated VT circuit activation time maps in a new righthand panel of Figure 4 (old 3).

- ❖ See throughout, particularly Introduction and Discussion.

Figure 2 has been squashed along the horizontal axis.

This figure has been wholly revised.

- ❖ See Figure 3 (old 2)

It would be helpful to include some information about the computational resources required to run the simulations, and in particular whether the workflow could be completed in a timescale that could be fast enough for the clinical setting.

Absolutely. This is a very important specific point that we have now included in the Methods.

- ❖ See Methods (ECG VT Simulation): "*The entire process …* "

Reviewer #2 (Remarks on code availability):

OpenCarp is a software tool, which can be downloaded and installed. It is well recognised in the community, and widely used. While the simulations described in the manuscript are in principle

reproducible, I could not find links to any of the anatomical models or specific metadata to run the simulations.

We entirely agree with the Reviewer on this point. As mentioned to the Editor, we have now uploaded representative anatomical bi-ventricular models for two representative patients, including universal coordinates and myofibre directions, along with the important wall thickness meta-data. This, combined with the detailed parameter file in the Supplement, should allow similar induction protocols to be executed in the OpenCARP software.

<u>Response to Editor's Comments</u>

No specific comments were made by the Editor other than to address the remaining comments from Reviewer 1 thoroughly and highlighting various manuscript formatting issues, both of which we have made every effort to address.

<u>Reviewer 1</u>

This reviewer appreciates the modification to the paper which has made it stronger however the revised paper still has some weaknesses that can be improved upon.

We thank the Reviewer for their appreciation of the previous improvements made to our paper, which we also agree significantly improved the study.

1. Figure 4. Shows Patient 8 has CC of 0.84 and patient 9 has CC of 0.83, however patient 8 has lack of concordance in leads V4, V5 and V6 whereas the text says Patient 9 only has lack of agreement on lead V4. These values does not seem to make sense. How can this be?

The Reviewer rightly highlights this important issue related to the specifics of how the correlation coefficient (CC) between simulated and clinical ECGs are computed. Firstly, it is important to note that the employed Pearson correlation coefficient is not dependent on signal amplitude, which can often lead to slight misinterpretation of numerical CC values from visual assessment of two plotted ECG traces (e.g. V6 in Patient 8 has a higher CC value than might be expected from visual assessment). Secondly, although leads LII, LIII, aVL & aVF in Patient 9 have matching polarities between simulated and clinical ECGs, we believe that the notching in the clinical traces have an important impact in CC which drives the mean value below that of Patient 8. Finally, as noted in the Methods, the reported values of CC relate to the mean of the top 10 (out of 12) traces, chosen to align more closely with clinical PaSo mapping strategies and which performed more robustly in our initial testing and method development.

We agree that these important issues were not adequately highlighted or discussed in the text and have now included additional text in the manuscript to reflect this.

- See Results, ECG Correlation Analysis of Simulated VT Circuits against clinical ECGs: "*matching polarities across … the mean CC value*".
- Figure 4 caption: "*Mean CC values … Quantitative ECG Comparison*."
- See Methods, Quantitative ECG Comparison: "*widely used in … relative signal amplitude*"

2. Figure 6. What weakens this figure is the lack of coregistration between the SATHE maps and what is maps on the bipolar map or VT LAT Map. In Patient 11 the area highlighted on the SATHe map does not correlate with the area on tbe bipolar and VT LAT map.

We agree with the Reviewer on the importance of adequately highlighting the correspondence between the SATHe Map predicted area and the clinical Electro-Anatomical Mapping data. The Reviewer rightly identifies that the match between the SATHe Map hot-spot and regions delineated as being at the periphery of dense scar in the bipolar match is indeed perhaps stronger in Patients 7 & 10 in the Figure. However, we do believe that, although not directly encompassing regions of intermediate voltage amplitude on the bipolar map, the hot-spot in Patient 11 still lies very close indeed to such regions. Related to the VT LAT map, we agree that interpretation of such maps can be rather subjective, but believe that the hot-spot region does align with a region in which early and late activated areas are in very close proximity (i.e. red-to-blue), whilst acknowledging that the match is perhaps closer in Patients 7 and 10. The issue of co-registration of imaging, modeling and electrophysiological data remains a key issue in all attempts to leverage pre-procedural data and we are grateful to the reviewer for highlighting the importance of this.

We acknowledge the importance of adequately describing these aspects, and have included additional text into the Results to address this.

- See Results, Comparison of SATHe Map Predictions with Clinical Measurements: "*Specifically, Patients 7 … all three patients.*" and "*demarcating characteristic transitions … in Patient 11.* ".
- See Figure 6, caption: "*characteristic of transitions … close spatial proximity.*"

What is the purpose of the maps with the ablation lesions? It does not seem the ablation lesions correlate what was mapped with the bipolar maps. If the ablation lesions were able to terminate the VT then why where they placed there and not where the SATHe Map suggested?

The purpose of showing the ablation lesions was to highlight the final substrate region targeted by the clinician in the procedure. These areas are often are quite dispersed, and it is also not possible to tell which were 'key' delivered lesions. The Reviewer rightly highlights that they do not necessarily align with the bipolar amplitude (or indeed VT LAT) maps. Although bipolar voltage provides some guidance to potentially relevant substrate to target, it is not the predominant substrate assessment strategy employed by the treating physician. Rather, a combination of voltage amplitude, signal complexity and regions of wavefront discontinuity are considered, in keeping with contemporary approaches to substrate based ablation for VT. In addition, VT specific data informs the ablation strategy including pace mapping, activation mapping and entrainment mapping where available.

These issues perhaps perfectly highlight the current problems with adequate target identification during VT ablation procedures, and the need for novel approaches. A prospective trial using our approach as targeted guidance is needed to fully assess ablation efficacy, which we are actively working towards. This initial work acts as essential retrospective validation, acknowledging the difficulties in exact identification of VT site-of-origin for ground truth in many cases.

We acknowledge the importance of these issues, and have added additional text to the manuscript to reflect this.

- See Results, Comparison of SATHe Map Predictions with Clinical Measurements: "*Note that in … substrate-based approach.*"

Figure 8 why is the shell registration in patient 5&8 poorly co-registered?

The Reviewer is right to raise the important issue related to registration between image-based guidance and electro-anatomic mapping (EAM) shells. In some patients, the EAM geometry does not extend quite as far in the basal or apical regions as the CT-based endocardium in the model, as the mapping catheter was not advanced fully into these areas during the procedure to accurately capture the entire endocardial surface geometry. Despite these slight differences in surface anatomy, we would like to assert that our registration methodology is consistent and we believe defensible as the optimal strategy currently available for this problem.

Specifically, our approach to registration of CT-based model and EAM shell does not require the acquisition of a comprehensive endocardial geometry. By employing fiducials derived from ultrasound (intra-cardiac echo), crucially acquired in the same image space as the EAM, robust and accurate registration with anatomical markers identified in the CT-based bi-ventricular model and the EAM itself to permit a registration approach independent of completeness of mapping of the endocardial surface. This is described in detail in Methods, Comparison with Clinical Data. We do acknowledge that registration remains a significant challenge that is likely to be a residual source of errors, despite our careful and thorough attempts to minimize these.

Due to the importance of this issue, we have included text in the Results acknowledging these registration considerations, and sign-posting more clearly to the description of our registration approach in the Methods.

- See Figure 6, caption: "*using ultrasound derived … with Clinical Data*"
- See Methods, Comparison with Clinical Data: "*Initial alignment was guided … endocardial map is obtained.*"

Reviewer #2

The revised manuscript is a much easier read. In particular Figure 1 is a great improvement, and makes the overall workflow much clearer to understand.

All of my comments on the initial draft have been addressed to my satisfaction..

We are pleased to hear that we addressed the Reviewer's previous concerns satisfactorily and thank the Reviewer once again for their positive appraisal of our work along with comments that helped us improve our manuscript.
